# Atomically Sharp 1D Interfaces in 2D Lateral Heterostructures of VSe_2_—NbSe_2_ Monolayers

**DOI:** 10.1021/acsnano.4c10302

**Published:** 2024-11-02

**Authors:** Xin Huang, Héctor González-Herrero, Orlando J. Silveira, Shawulienu Kezilebieke, Peter Liljeroth, Jani Sainio

**Affiliations:** † Department of Applied Physics, Aalto University, FI-00076 Aalto, Finland; ‡ Departamento Física de la Materia Condensada, Universidad Autónoma de Madrid, Madrid E-28049, Spain; § Department of Physics, Department of Chemistry and Nanoscience Center, University of Jyväskylä, FI-40014 Jyväskylä, Finland

**Keywords:** TMDC, lateral heterostructure, STM, DFT, MBE

## Abstract

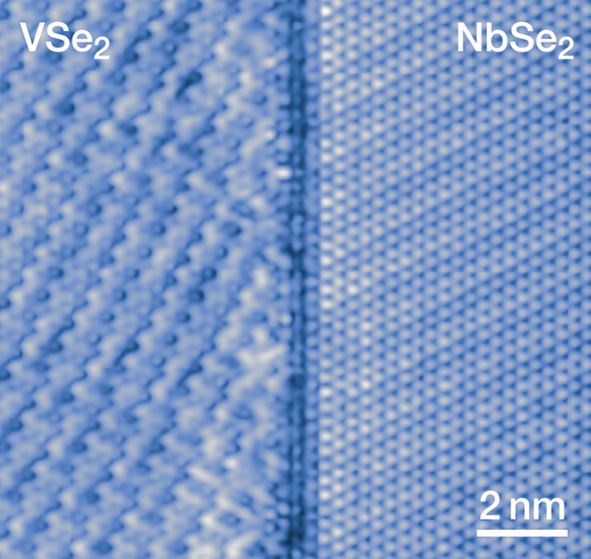

van der Waals heterostructures have emerged as an ideal platform for creating engineered artificial electronic states. While vertical heterostructures have been extensively studied, realizing high-quality lateral heterostructures with atomically sharp interfaces remains a major experimental challenge. Here, we advance a one-pot two-step molecular beam lateral epitaxy approach and successfully synthesize atomically well-defined 1T-VSe_2_—1H-NbSe_2_ lateral heterostructures. We demonstrate the formation of defect-free lateral heterostructures and characterize their electronic structure by using scanning tunneling microscopy and spectroscopy together with density functional theory calculations. We find additional electronic states at the 1D interface as well as signatures of Kondo resonances in a side-coupled geometry. Our experiments explored the full potential of lateral heterostructures for realizing exotic electronic states in low-dimensional systems for further studies of artificial designer quantum materials.

Heterostructures of two-dimensional (2D) materials are seen as one of the most flexible platforms to study correlated electronic states and realize intricate phenomena in condensed matter systems.^1−3^ Most van der Waals (vdW) heterostructures are assembled through vertical stacking, where layers interact only via van der Waals forces. These vertical heterostructures de facto realize an effective 2D system. In addition to these 2D systems, it would be desirable to have access to one-dimensional (1D) structures, where different electronic phenomena can arise. 1D lattices with a lower dimensional structure also provide a simpler prototype to understand many-body physics. However, dimensionality reduction starting from a higher dimension remains challenging; it is inherently difficult to fabricate 1D structures with top-down methods, e.g., mechanical exfoliation and transfer. Currently, almost all experimentally realized 1D structures in 2D materials are naturally occurring, for example, grain boundaries or domain walls.^4−7^

On the other hand, fabricating lateral heterostructures by bottom-up synthesis offers intriguing possibilities for creating 1D structures. Compared to their vertical counterparts, lateral heterostructures or in-plane heterojunctions have covalent bonds between the components and can form artificial 1D structures capable of hosting exotic electronic states. However, the most common method to produce lateral heterostructures—chemical vapor deposition (CVD)—has considerable drawbacks: the atomic-scale structure of CVD-grown interfaces typically suffers from a high density of imperfections, such as elemental doping and alloying, various defects, and dislocations.^8−19^ In addition, most attention on lateral heterostructures in transition metal dichalcogenides (TMDCs) to date has focused on homophase semiconductor-semiconductor junctions (e.g., MoS_2_, MoSe_2_, WS_2_, and WSe_2_), where the two components have the same crystal structure, e.g., 1H- with 1H-phase.^8−16,20,21^

In this work, we choose two heterophase metallic TMDC monolayers, vanadium diselenide (VSe_2_) and niobium diselenide (NbSe_2_), with different crystal phases of 1T and 1H with both having electronic structures where electron correlations play a significant role. VSe_2_ is metallic in its monolayer octahedral 1T structure, and it has been reported to have various possible magnetic ground states competing with charge density wave (CDW) order depending on factors such as defect density, doping, and strain.^22−30^ The other ingredient, 1H-phase of NbSe_2_, is a 2D metal exhibiting significant electron correlations, and CDW and superconducting orders at low temperatures (superconducting *T*_c_ ∼ 1 K for a monolayer).^31−37^

Here, we demonstrate a one-pot, two-step lateral epitaxy technique to fabricate atomically sharp and well-defined lateral heterostructures of 1T-VSe_2_—1H-NbSe_2_ by molecular beam epitaxy (MBE). We probe them by low-temperature scanning tunneling microscopy (STM) and spectroscopy (STS) and identify two different 1D interface structures corroborated by density functional theory (DFT) calculations. These heterostructures exhibit 1D interfacial states and signatures of Kondo resonances in an atomic-scale side-coupled geometry. This work demonstrates an approach for achieving complex lateral heterostructures with atomically well-defined 1D interfaces where it is possible to realize correlated many-body states via lateral coupling.

## Results and Discussion

### Synthesis and Structure of the Lateral Heterostructures

**Figure 1:**
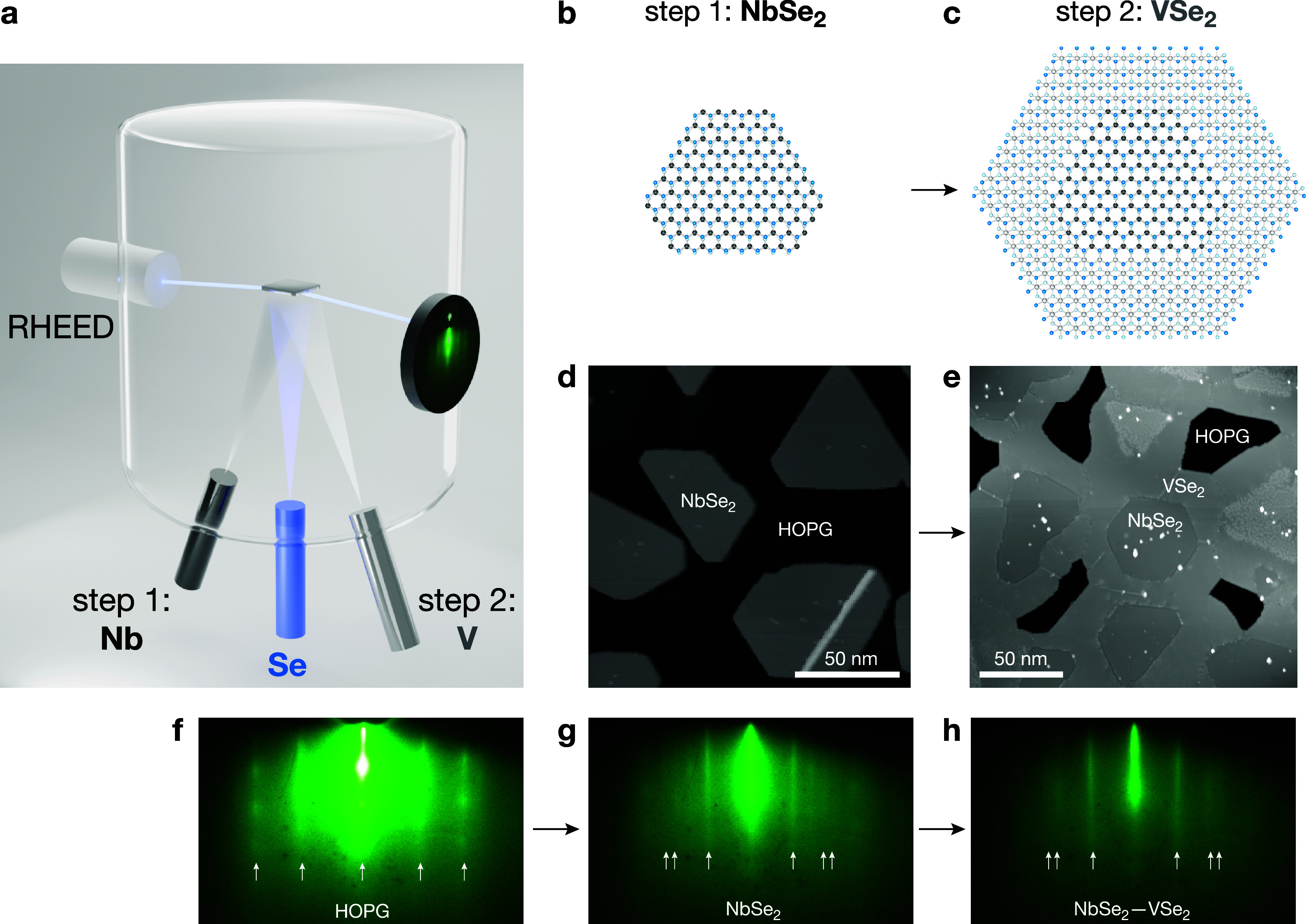
Synthesis of the lateral heterostructures. (a) Illustration of our one-pot, two-step lateral epitaxy. (b) Schematic of a NbSe_2_ island and (c) in-registry lateral epitaxy of VSe_2_ surrounding the NbSe_2_ island. (d) Scanning tunneling microscopy (STM) topography image of the sample after step 1: growth of NbSe_2_. *V*_s_ = +1 V, *I*_t_ = 2 pA. (e) STM image after step 2: growth of VSe_2_. *V*_s_ = −1.5 V, *I*_t_ = 10 pA. (f–h) Reflection high-energy electron diffraction (RHEED) patterns of the substrate HOPG (f), monolayer NbSe_2_ islands (step 1, g), and VSe_2_—NbSe_2_ lateral heterostructures (step 2, h).

We overcome the challenges in CVD^8−16,20^ by introducing a one-pot two-step lateral epitaxy method utilizing MBE (Figure 1 and Methods). The first step is to grow monolayer islands of 1H-phase NbSe_2_ at 550 °C with well-defined and straight edges (Figure 1b,d). The second step is the lateral epitaxy growth of 1T-VSe_2_ at 375 °C (Figure 1c,e and Supplementary Figures S1 and S2), using the 1H-NbSe_2_ islands’ edges as seeds.^38,39^ Growth temperature and growth sequence play a crucial role^40^ in lateral epitaxy: the material with higher growth temperature should be synthesized first in order to preserve island morphology and the edge structure and to prevent unwanted alloying in the next steps. Our molecular beam lateral epitaxy growth can be monitored in situ via reflection high-energy electron diffraction (RHEED). During step 1, the RHEED pattern gradually develops stripes of NbSe_2_ in addition to the pattern related to the highly oriented pyrolytic graphite (HOPG) substrate (Figure 1f,g). Subsequently, during step 2, the RHEED pattern of the newly formed lateral heterostructure basically overlaps with the one of the NbSe_2_, because the nominal lattice constant of VSe_2_ is very similar to that of NbSe_2_ (∼0.1 Å difference) and the overall increasing coverage of the monolayer leads to dimming and the eventual disappearance of the HOPG RHEED pattern (Figure 1h). We have also calculated the lattice constants from RHEED: for 1T-VSe_2_, we obtained 3.36 ± 0.09 Å (Supplementary Figure S3), for 1H-NbSe_2_ 3.46 ± 0.09 Å, and for the NbSe_2_–VSe_2_ heterostructure 3.38 ± 0.09 Å.

**Figure 2:**
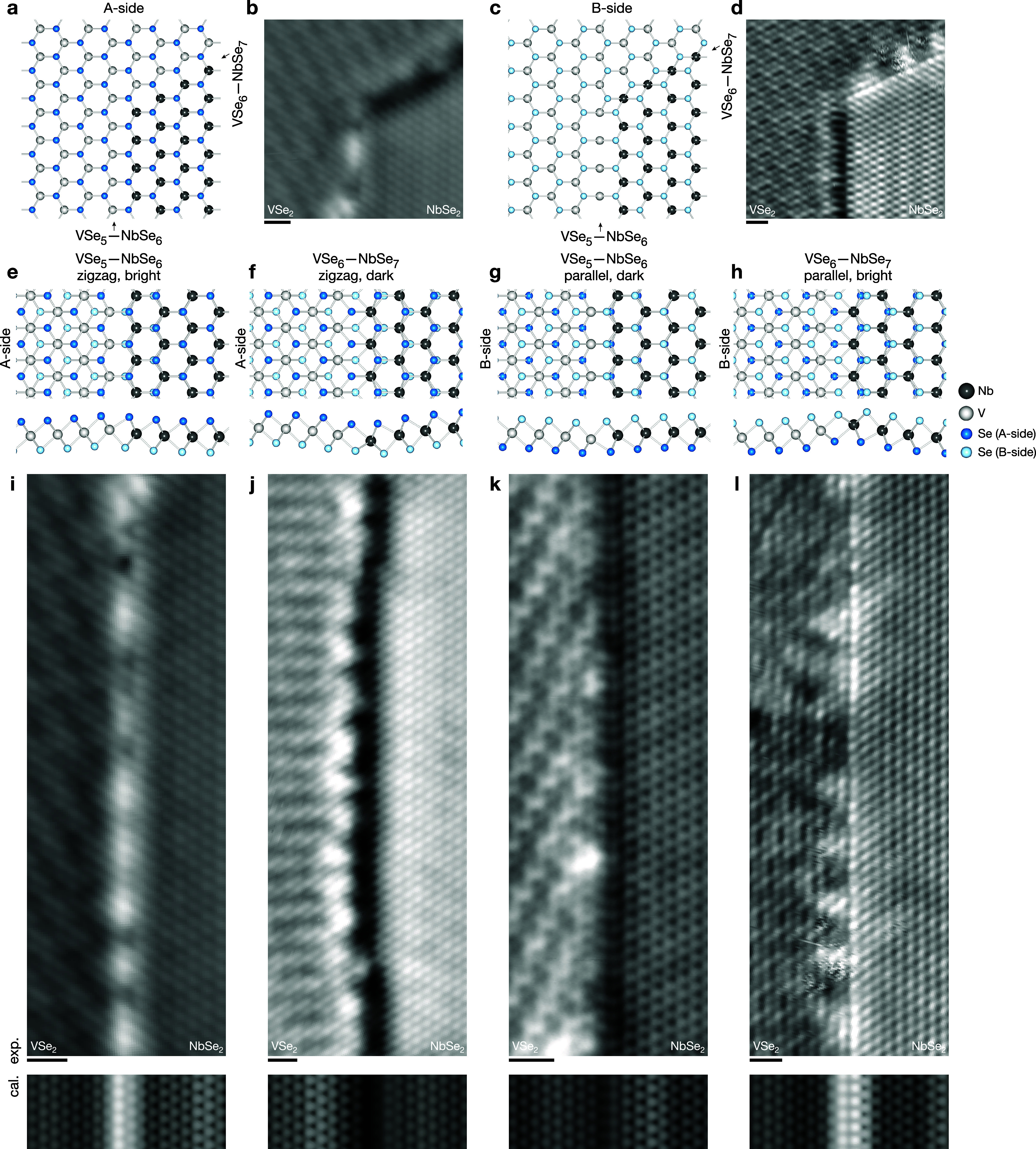
Two types of lateral heterostructures. (a) A-side view (with A-side Se atoms only) and (b) its STM topography image. (c) B-side view (with B-side Se atoms only) and (d) its STM topography image. (e–h) Top and side view of the two types of lateral heterostructures and (i–l) their corresponding atomically resolved STM topography images (experimental and calculated). Scan parameters: (b, d, i, j, l) *V*_s_ = −1 V, *I*_t_ = 100 pA. (k) *V*_s_ = −0.99 V, *I*_t_ = 50 pA. Calculated STM images, *V*_s_=–0.5 V. All scale bars are 1 nm.

We confirm the growth of 1H-NbSe_2_ and 1T-VSe_2_ by their STM topography images with typical charge density waves [3 × 3 for 1H-NbSe_2_,^31^ and  for 1T-VSe_2_^23,29,41^ (see Supplementary Figures S2, S5 and S6)]. Creating lateral heterostructures from such systems provides opportunities for research on 2D charge density wave orders. In our heterostructures, the intrinsic CDWs of both materials extend right up to the interface (Figure 2 and Supplementary Figures S5–S10), where they abruptly switch from the characteristic CDW of 1H-NbSe_2_ to that of 1T-VSe_2_ in contrast to a similar system showing a CDW proximity effect.^37^ The undeformed CDWs indicate that there is no observable in-plane lattice distortion, implying a lack of significant amounts of, e.g., strain or doping being induced at the interface, as especially the CDW in 1T-VSe_2_ should be sensitive to those.^26^

We find two different types of lateral heterostructures, as shown in Figure 2. The 1D interfaces are atomically sharp without cross-contamination, doping, or alloying, with the length of the straight sections extending up to ∼20 to 40 nm (Figure 2 and Supplementary Figures S6–S9), which to our knowledge are the longest high-quality TMDC lateral heterostructures grown so far.^19^ The first-grown 1H-NbSe_2_ islands mostly have a hexagonal shape with 120° corners. Considering the crystal structure, this means that the neighboring edges are crystallographically distinct, i.e., with alternating edge terminations with e.g., Nb- or Se-terminated edges.^42,43^ The subsequent epitaxy of 1T-VSe_2_ will most likely form Nb–Se–V chemical bonds over the interface, especially in a Se-rich growth environment. Combining this with the 1T-VSe_2_ crystal structure, we can identify four different types of possible lateral heterostructures, with vanadium and niobium coordination numbers between 5 and 7 (Supplementary Figures S16 and S18). Corroborated by our DFT calculations, we identify the two most stable heterostructures, and we label them as VSe_5_—NbSe_6_ and VSe_6_—NbSe_7_ (Figure 2a,c). Also, other types of structures have been considered, but they either fail to produce the correct type of atomic arrangement seen in Figure 2 or are inconsistent with in-registry growth around 120° corners of 1H-NbSe_2_ islands (Supplementary Figure S16).

However, depending on which side of these structures grows on the substrate (and hence which side faces the STM tip), these two types of lateral heterostructures show four different STM topographies (arising mostly from the top layer of Se atoms), as shown in Figure 2e–h and labeled zigzag or parallel with bright or dark contrast. Hence, the two different interface structures, VSe_5_—NbSe_6_ and VSe_6_—NbSe_7_, can appear in two different orientations on the surface corresponding to different sides of the monolayer, labeled A-side and B-side. If imaged on the A-side, the VSe_5_—NbSe_6_ interface shows a bright zigzag structure and VSe_6_—NbSe_7_ a dark zigzag; if scanning on the B-side, VSe_5_—NbSe_6_ shows a dark parallel structure and VSe_6_—NbSe_7_ a bright parallel one.

Our DFT calculations also give the correct contrast from the top layer Se atoms in simulated STM images compared to the experimental ones (Figure 2i–l). The DFT calculations suggest that the interfaces have a slight structural deformation producing a structure similar to that of the 1T*′*-phase, and the trend of bright or dark contrast comes in part from a small corrugation at the interface (Figure 2e–h). The contrast also has an electronic component discussed later. We find that the bright and dark contrasts alternate between the adjacent edges of hexagonal 1H-NbSe_2_ islands (see Figure 2b,d, and Supplementary Figures S6–S9). In addition, interfaces around a single 1H-NbSe_2_ island are either zigzag or all parallel. These two experimental observations further support the structural assignment above and are consistent with our DFT calculations (Figure 2e–h).

### Electronic Structure of the 1D Interfaces

**Figure 3:**
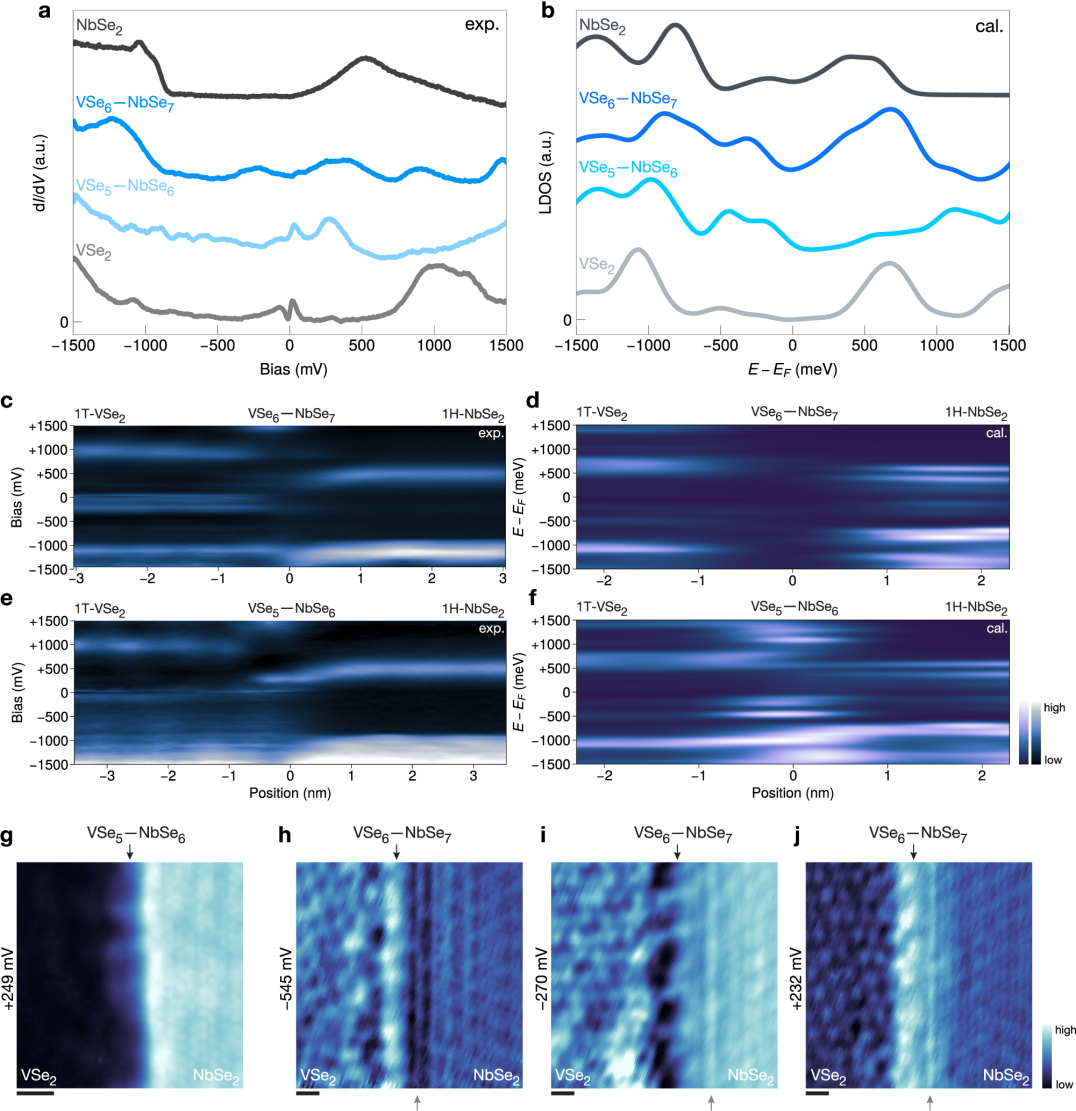
Electronic structure of the lateral heterostructures. (a) d*I*/d*V* spectra of 1H-NbSe_2_, 1T-VSe2_2_, VSe_5_—NbSe_6_, and VSe_6_—NbSe_7_ interfaces (shifted vertically for clarity). (b) DFT-calculated LDOS of 1H-NbSe_2_, 1T-VSe2_2_, VSe_5_—NbSe_6_, and VSe_6_—NbSe_7_ interfaces (shifted vertically for clarity). (c–f) Experimental d*I*/d*V* spectra and calculated LDOS along a line across the VSe_6_—NbSe_7_ (panels c and d) and VSe_5_—NbSe_6_ (panels e and f) interfaces. (g) d*I*/d*V* map of the 1D interfacial state of VSe_5_—NbSe_6_, at +249 mV (*V*_mod_ = 4 mV). (h–j) Constant-current d*I*/d*V* maps of VSe_6_—NbSe_7_ interface at −545, −270, and +232 mV, respectively (*V*_mod_ = 10 mV), which show additional line–like LDOS modulation inside 1H-NbSe_2_ in the vicinity of the VSe_6_—NbSe_7_ interface. Top black arrows indicate the interfaces, while bottom gray arrows mark the modulation features. All scale bars are 1 nm.

We focus next on the electronic behavior of the two lateral heterostructures on the A-side with zigzag interfaces (Figure 3). The differential conductance (d*I*/d*V*) spectrum of monolayer 1H-NbSe_2_ shows a peak at around +500 mV related to the Nb-based conduction band, and the peak ∼−1100 mV is related to its valence band.^31,44^ The conduction band crosses the Fermi level away from the Γ-point, and it is difficult to resolve the bottom of the conduction band.^31^ Together with the gap between the bottom of the conduction band and the valence band, this results in a bias region with a low d*I*/d*V* signal (from ∼0 mV to ∼− 840 mV). The d*I*/d*V* of 1T-VSe_2_ is consistent with the results reported in the literature,^24,28,45,46^ with the signal close to the Fermi level arising from the vanadium *d*-states and V-shaped features very close to *E*_F_ resulting from the CDW.^24,46^

The two interfaces show quite different features compared with each other and the corresponding 1H-NbSe_2_ and 1T-VSe_2_ bulk monolayers. For the VSe_5_—NbSe_6_ interface, a prominent peak shows up around +250 mV (Figure 3a). From the differential conductance (d*I*/d*V*) map taken at this energy (Figure 3g), we can confirm that this state is localized at the interface, with a spatial extent of roughly 1 nm. For VSe_6_—NbSe_7_, no additional states are observed but the Nb-based conduction band shifts to lower energy very close to the interface while the valence band of 1T-VSe_2_ remains at the same energy. The evolution of the electronic states can be also visualized by recording spectra along a line across the interface, as shown in Figure 3c,e.

DFT-calculated LDOS spectra (Figure 3b,d,f, and Supplementary Figure S15) show similar behavior. The additional electronic state of VSe_5_—NbSe_6_ is reproduced in the calculated LDOS (with an energy shift which is also seen for the monolayer 1T-VSe_2_ states). This state is absent at the VSe_6_—NbSe_7_ interface.

We also calculated the projected density of states (PDOS) on the top layer Se atoms at the VSe_5_—NbSe_6_ interface (see Supplementary Figure S17), since they contribute most to the STM signal. The calculations indicate that the bright contrast at the interface is due not only to the corrugation of the Se atoms but also to changes in the electronic structure. The same is true for the VSe_6_—NbSe_7_ interface (dark contrast), but in that case, the effect of the corrugation appears to be larger.

Finally, we observed additional density of states oscillations parallel to the VSe_6_—NbSe_7_ interface on the 1H-NbSe_2_ side, in addition to its normal 3 × 3 CDW (Figure 3h–j, the constant-current d*I*/d*V* maps). This additional modulation could arise from interference of incident and elastically scattered CDWs or from Friedel oscillations^43,47,48^ and it is absent on the VSe_5_—NbSe_6_ interfaces.

### Signatures of Kondo Resonances in a Side-Coupled Geometry at the Interfaces

**Figure 4:**
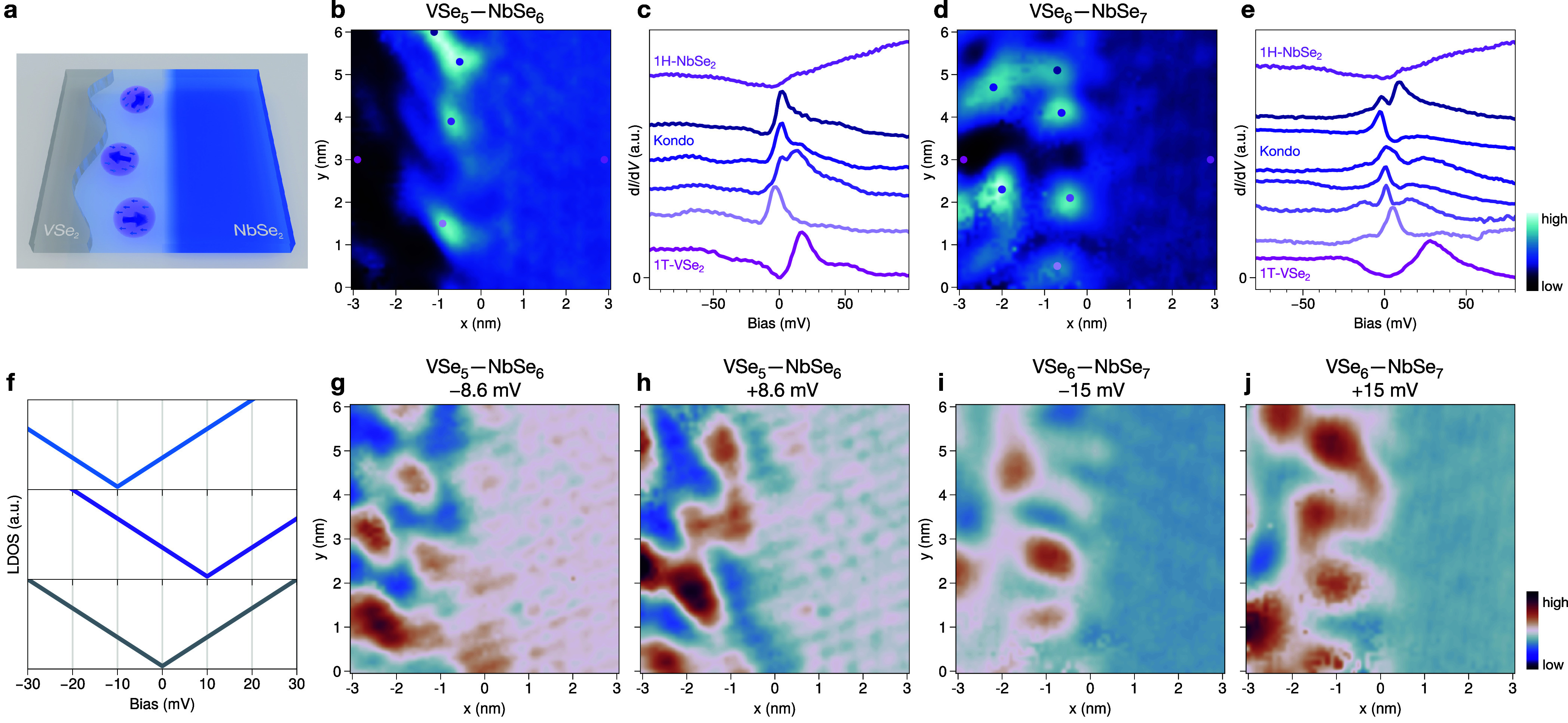
Signatures of the Kondo effect in a side-coupled geometry and contrast inversion near the interfaces. (a) Schematic of Kondo singlets in a side-coupled geometry. Conduction electrons (light blue area) screen localized moments (purple arrows), forming Kondo singlets. (b) d*I*/d*V* map at −0.8 mV of VSe_5_—NbSe_6_ interfaces, *V*_mod_ = 2 mV. (c) Point d*I*/d*V* spectra from positions marked in (b) (shifted vertically for clarity). (d) d*I*/d*V* map at 0 mV of VSe_6_—NbSe_7_ interfaces, *V*_mod_ = 1 mV. (e) Point d*I*/d*V* spectra from positions marked in (d) (shifted vertically for clarity). (f) Schematic of the V-shaped LDOS around the Fermi level of different spots inside 1T-VSe_2_: negatively charged (top), positively charged (middle), and neutral without charge doping (bottom). (g, h) Contrast inversion of a d*I*/d*V* map of VSe_5_—NbSe_6_ at ±8.6 mV, *V*_mod_ = 2 mV. (i, j) Contrast inversion of a d*I*/d*V* map of VSe_6_—NbSe_7_ at ±15 mV, *V*_mod_ = 1 mV.

Lateral heterostructures provide an ideal platform to create 1D interfaces that combine properties of different materials and allow for direct visualization of the created designer quantum state in real space. Our heteroepitaxy protocol ensures atomically sharp interfaces, largely eliminating the undesirable interference from imperfections (e.g., defect states). Here, we give one demonstration of such designer phenomena in our artificial composite materials with side-coupled geometry. In addition to the electronic effects discussed in the previous section, we observe both interfaces exhibiting strong localized zero bias anomalies with typical peak or dip-peak features (Figure 4c,e and Supplementary Figures S11 and S12). At the same time, our STS lacks typical inelastic tunneling features that could be associated with spin-flip or magnon excitations.^49−51^ While not conclusively proven by, e.g., temperature and magnetic field dependence, we consider the most likely explanation for these zero bias anomalies to be Kondo resonances arising from coupling between localized magnetic moments in 1T-VSe_2_ and conduction electrons in 1H-NbSe_2_ (Figure 4a). These Kondo resonances are unlikely to arise from the coupling between 1T-VSe_2_ and the substrate HOPG, since zero bias anomalies in pristine 1T-VSe_2_ are absent in our results (Figure 4c,e and Supplementary Figures S11 and S12) and other reports on the pure monolayer 1T-VSe_2_/HOPG system.^24,52^ The emergence of free magnetic moments in 1T-VSe_2_ could be related to the ground state of the material itself, or to an interfacial effect, with contributions from, e.g., inhomogeneous strain and charge transfer. Similar Kondo resonances have been previously observed close to the edges of 1T-VSe_2_ islands on bulk 2H-NbSe_2_.^28^ Thus, it is most likely that 1H-NbSe_2_ acts as the electron bath/charge reservoir, which couples to the localized magnetic moments inside 1T-VSe_2_.^24,28,53,54^ These lateral heterostructures realize Kondo resonances in a side-coupled geometry (Figure 4a), which has been previously reported only in mesoscopic side-coupled quantum dot experiments.^55−57^

The maximum intensity of the Kondo signal is found inside 1T-VSe_2_ rather than exactly at the interface (Figure 4b,d). They can extend in some cases even up to ∼8 nm away from the interface to the VSe_2_ side (see Supplementary Figure S12). Besides the appearance of localized moments in VSe_2_, the formation of these Kondo singlets depends on the possible spacial range of screening by the conduction electrons of NbSe_2_. The positions of Kondo sites show real-space modulation, but they are not directly linked with the periodicity of either the VSe_2_ lattice or the CDW in 1T-VSe_2_ or 1H-NbSe_2_ (Figure 4b,d), which could be related to the inhomogeneous charge distribution discussed below. This, taken together with the signature of Kondo resonances, suggests a lack of magnetic order in 1T-VSe_2_ around the interfaces.

For 1T-VSe_2_ near the interface, we also observe a contrast inversion in differential conductance (d*I*/d*V*) maps at small positive and negative bias, at energies inside the V-shaped local density of states (LDOS) (see Figure 4f–j and Supplementary Figure S12). We attribute this phenomenon to a spontaneous inhomogeneous electronic charge redistribution in 1T-VSe_2_. Hence, at different positions, the electronic doping shifts the 1T-VSe_2_’s V-shaped LDOS toward positive/negative energy, as suggested in Figure 4f. When obtaining d*I*/d*V* maps, e.g., at positive bias +10 mV, the negatively charged spots get brighter contrast, while positively charged spots get darker contrast; and when taking at opposite bias, the contrast of d*I*/d*V* maps reverses.^58,59^ In comparison, 1H-NbSe_2_ is basically free from contrast inversion. We speculate that this spontaneous charge redistribution may be a feature of 1T-VSe_2_ itself, or a result of charge transfer to/from 1H-NbSe_2_, which would depend on the CDW periodicity of both 1H-NbSe_2_ and 1T-VSe_2_, and thus could be quite inhomogeneous.

## Conclusions

In this work, we introduce a one-pot, two-step heteroepitaxy method for constructing atomically sharp 1T-VSe_2_—1H-NbSe_2_ lateral heterostructures. We systematically study these defect-free, straight 1D interfaces to reveal their atomic-level geometric and electronic structures using STM and STS experiments corroborated by DFT calculations. We identify two structures, VSe_5_—NbSe_6_ and VSe_6_—NbSe_7_, and find electronic states and charge modulation localized at or near the 1D interfaces. We demonstrate that these types of lateral heterostructures can be used to realize exotic electronic states, in our case Kondo resonances, arising from the coupling of the magnetic moments of 1T-VSe_2_ with 1H-NbSe_2_ conduction electrons. Our work presents a general method for constructing atomically perfect 1D interfaces in TMDC lateral heterostructures for further studies of correlated 1D systems.

## Methods

### Synthesis of Heterostructures

We use an enhanced MBE protocol (one-pot two-step molecular beam lateral epitaxy) to synthesize VSe_2_, NbSe_2,_ and their lateral heterostructures in ultrahigh vacuum (UHV) (base pressure of ∼8 × 10^–9^ mbar). Vanadium rod (99.8%, Goodfellow Cambridge Ltd.) and niobium rod (99.9%, MaTecK GmbH) were evaporated by electron-beam heating (EFM 3T, Focus GmbH). Se powder (99.99%, Sigma-Aldrich) was evaporated in an effusion cell at ∼140 °C with a thermal cracker at ∼1200 °C (Thermal cracker cell, MBE-Komponenten GmbH). All samples were grown on highly oriented pyrolytic graphite (HOPG) (ZYB grade, TipsNano Co.), which were previously degassed above ∼600 °C. The VSe_2_—NbSe_2_ lateral heterostructure was synthesized in two steps: first, growing NbSe_2_ at ∼550 °C with a growth rate of 29 min per monolayer, with 30 min post annealing at ∼400 °C; second, growing VSe_2_ at a substrate temperature of ∼375 °C with a growth rate of 14 min per monolayer, with 5 min post annealing at ∼375 °C. Later, to protect materials during transferring to STM, samples were capped in Se vapor with an amorphous Se layer (>10 nm).

### STM Measurements

The STM experiments were carried out in another UHV setup following removal of the selenium capping by gentle thermal annealing of the samples (between 250 and 300 °C) for 1 to 2 h. This confirms the stability of our lateral heterostructures up to these temperatures. All the STM images and spectra were acquired at ∼4.2 K using a Createc LT-STM (CreaTec GmbH), or Unisoku USM-1300 (Unisoku Co., Ltd.) at 2 K (Figure 1d). For STM topography images, feedback set-point bias voltage (*V*_s_) and tunneling current (*I*_t_) are given in the figure captions. Topography images are rendered with Gwyddion.^60^ Scanning tunneling spectra (STS) or differential conductance (d*I*/d*V*) spectroscopy are measured with the lock-in technique at the frequency of 746 Hz, and the peak-to-peak modulation voltage (*V*_mod_) is specified in the figure caption.

### Density Functional Theory Calculations

DFT calculations were performed with the *QUANTUM ESPRESSO* distribution.^61^ Interaction between electrons and ions were described with the PAW pseudopotentials,^62,63^ while the electronic wave functions were expanded considering a plane-wave basis set with kinetic energy cutoffs of 90 Ry. For the lateral heterostructures, the integration over the Brillouin zone (BZ) was performed using a uniform grid of 1 × 8 × 1 k-point. For the results shown in the main paper, we have adopted the standard Perdew–Burke–Ernzerhof (PBE) functional augmented with + U correction of 2 eV on V-3d orbitals.^64,65^ Results with other values of + U are present in the SI. STM simulations, and LDOS maps were obtained with the *critic2* code.^66,67^

## Supporting Information Available

Additional STM data on growth of heterostructures, CDW orientations and Kondo resonances, additional RHEED data including lattice constant determination, and detailed DFT calculations (PDF)
