# Supplementary material for: Atomically Sharp 1D Interfaces in 2D Lateral Heterostructures of VSe2—NbSe2 Monolayers
Source: ACS Nano. 2024 Nov 2;18(45):31300–8. doi: 10.1021/acsnano.4c10302 (PMC11562791; doi:10.1021/acsnano.4c10302)
Supplement: Supplementary file 1 — nn4c10302_si_001.pdf [file nn4c10302_si_001.pdf]

Supporting Information: Atomically sharp 1D interfaces in 2D lateral heterostructures of VSe_2—$NbSe_2$ monolayers

Xin Huang,[†,§] Héctor González-Herrero,[†,‡,§] Orlando J. Silveira,[†] Shawulienu Kezilebieke,[¶] Peter Liljeroth,[†] and Jani Sainio[*,†]

†*Department of Applied Physics, Aalto University, FI-00076 Aalto, Finland*

‡*Departamento Física de la Materia Condensada, Universidad Autónoma de Madrid, Madrid E-28049, Spain*

¶*Department of Physics, Department of Chemistry and Nanoscience Center, University of Jyväskylä, FI-40014 University of Jyväskylä, Finland*

§*These two authors contributed equally*

E-mail: jani.sainio@aalto.fi

Experimental section

Figure S1 shows height data for our VSe_2–$NbSe_2$ heterostructures indicating that our lateral structures are monolayers. The obtained apparent height is 0.92 nm for the 1T-VSe_2 layer and 0.90 nm for the 1H-$NbSe_2$ layer. These values are in good agreement with other STM studies of VSe_2/HOPG: 0.85 nm;[S1] $NbSe_2$/HOPG (or graphene): about 0.7 nm[S2,S3] keeping in mind that the apparent heights depend on the bias set-point and only roughly reflect the actual height of the layers. The lateral interface between VSe_2 and $NbSe_2$ is found to be very smooth with a height difference of only 20 pm. Fig. S2 shows large area topographic data on our heterostructures as well as typical charge density waves of individual areas of the sample. From the topographic data one can observe that 1H-$NbSe_2$ islands have preferred growth directions on HOPG. Upon closer examination one finds two preferred orientations of 1H-$NbSe_2$ islands with a 30° rotation between them (also seen in main text Fig. 1).

Figures S3 and S4 (and main text Figure 1) show RHEED patterns of 1T-VSe_2, 1H-$NbSe_2$ and the 1T-VSe_2—1H-$NbSe_2$ heterostructure. The RHEED patterns do not show constant streak distances. This indicates the presence of rotational domains which occur naturally in HOPG.[S4] In addition, the previously mentioned two preferred growth directions of $NbSe_2$ will produce the same effect in the RHEED pattern. We have determined the lattice constants from these RHEED patters as indicated in Figure S4 by measuring the streak distances with respect to HOPG.

Next we focus on the interfaces of the 1T-VSe_2—1H-$NbSe_2$ lateral heterostructures. Figure S5 shows a STM image of one interface with fast Fourier transforms (FFTs) of areas near the interface on both sides. Figure S5c is the FFT of 1T-VSe_2 with exhibits typical 1×1 spots (circled in yellow) and CDW related spots (circled in blue). Figure S5e shows the FFT of 1H-$NbSe_2$ with its typical 3×3 CDW. The atomic resolution STM images and their FFT both support the observation that the two materials retain their regular structure right up to the interface.

For 1T-VSe_2, we find different CDW orientations with respect to the interfaces where

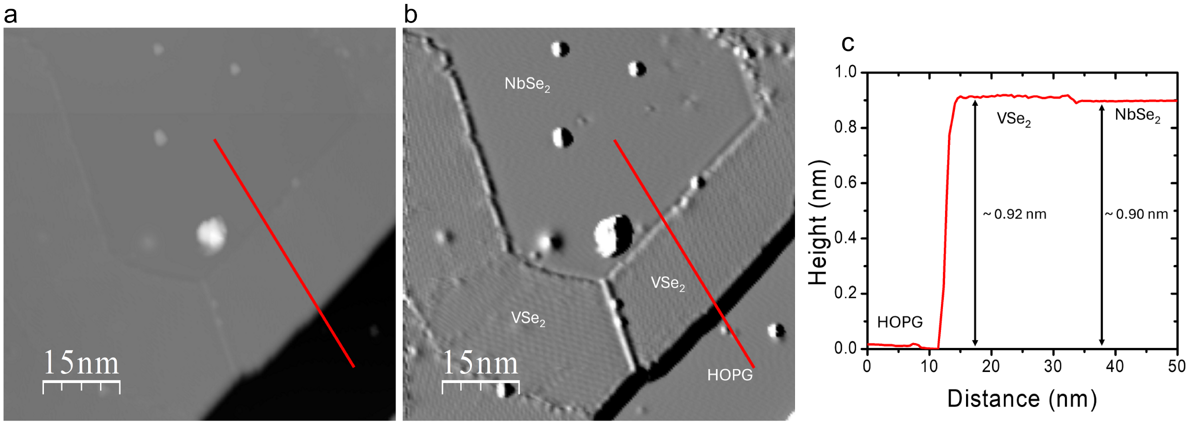

Fig. S1: Monolayer VSe_2 and $NbSe_2$ height data. **a**, STM image of a region of our VSe_2-$NbSe_2$ lateral heterostructures sample. (V_S=-1.5V; I_t=10pA). **b**, Same image as panel **a** where a derivative filter has been used to better differentiate the VSe_2 and $NbSe_2$ regions on the image. **c**, Height profile along the red line marked in panels **a** and **b** showing the height of VSe_2 and $NbSe_2$ monolayers.

the CDW "stripes" are approximately perpendicular to the interface or at an angle of 30°/150° (see Figs. S5–S10). Thus, there seems to be a preferred nucleation direction for the 1T-VSe_2 CDW and the observed angles are only a result of the spreading of the VSe_2 layer to other edges/islands. As mentioned before, there can be two growth orientations for 1H-$NbSe_2$ which will add more complexity to the possible CDW directions. In areas where rotated domains of the 1T-VSe_2 CDW meet we observe grain boundaries, as seen *e.g.* in Figs. S7 and S9. For 1H-$NbSe_2$ we occasionally observe stripe–like areas of the CDW in stead of the normal 3×3 structure (see *e.g.* Fig. S6). These have been previously shown to be caused by even small amounts of strain.[S5–S7] However, since we mostly observe areas with unperturbed 3×3 CDW contrast, the strain would not seem to be uniform throughout the $NbSe_2$ islands.

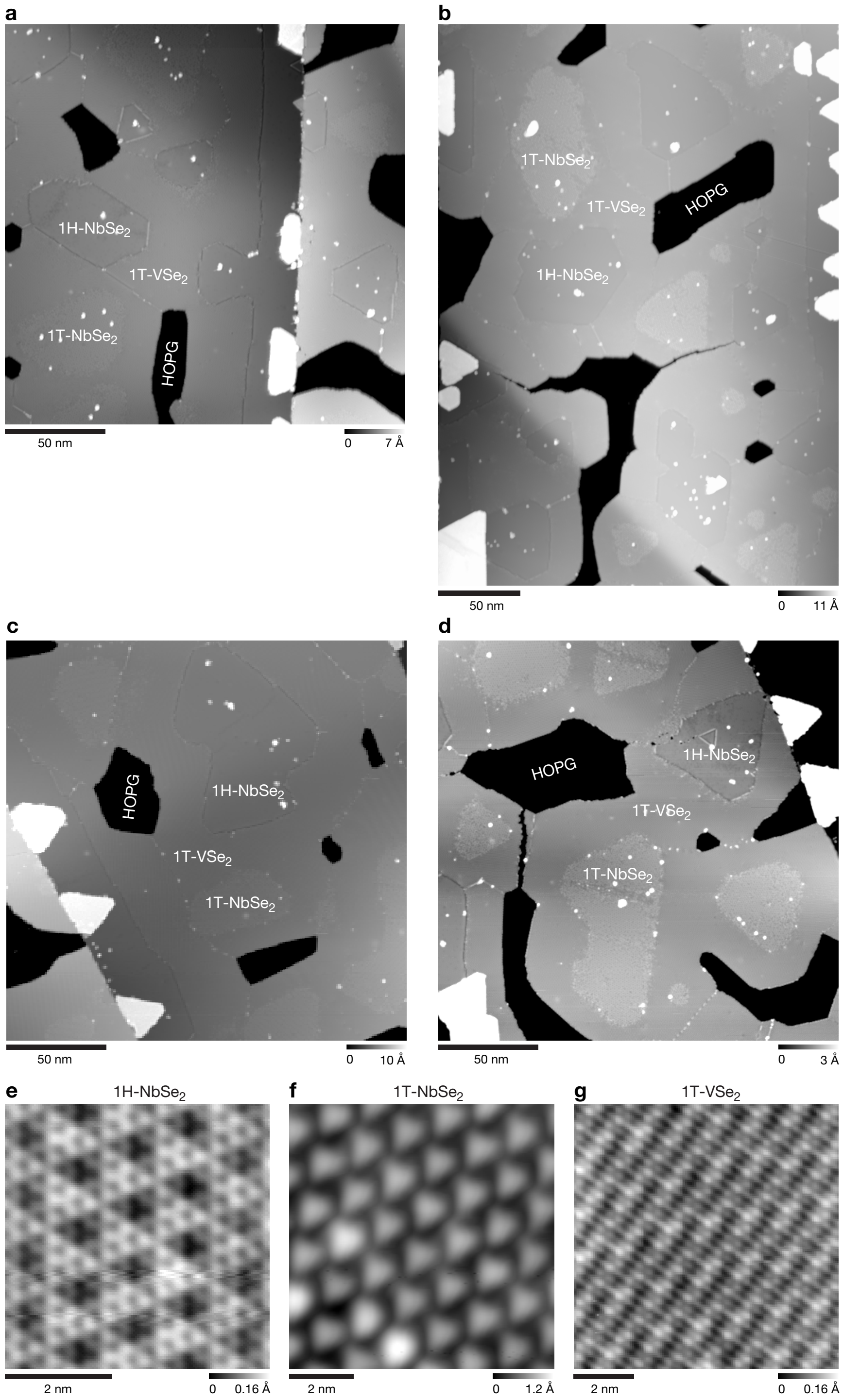

Fig. S2: Lateral heteroepitaxy protocol: VSe_2 preferentially grows laterally from the edges of $NbSe_2$ islands, rather than exhibting vertical growth. During the $NbSe_2$ growth step, both phases 1H- and 1T-$NbSe_2$ can be found and consequently, we form lateral heterostructures of the two $NbSe_2$ phases with 1T-VSe_2. **a**–**d**, Large-scale STM topography images of several different regions of the sample. Typical charge density wave of **e**, 1H-$NbSe_2$; **f**, 1T-$NbSe_2$; **g**, 1T-VSe_2, from STM topography image. Scan parameters: **a**, V_s=+1.36 V, I_t=10 pA. **b**, V_s=−1.5 V, I_t=10 pA. **c**, V_s=+1.541 V, I_t=6.5 pA. **d**, V_s=+1.5 V, I_t=4.1 pA. **e**, V_s=−0.36 V, I_t=100 pA. **f**, V_s=−0.649 V, I_t=400 pA. **g**, V_s=−1 V, I_t=100 pA.

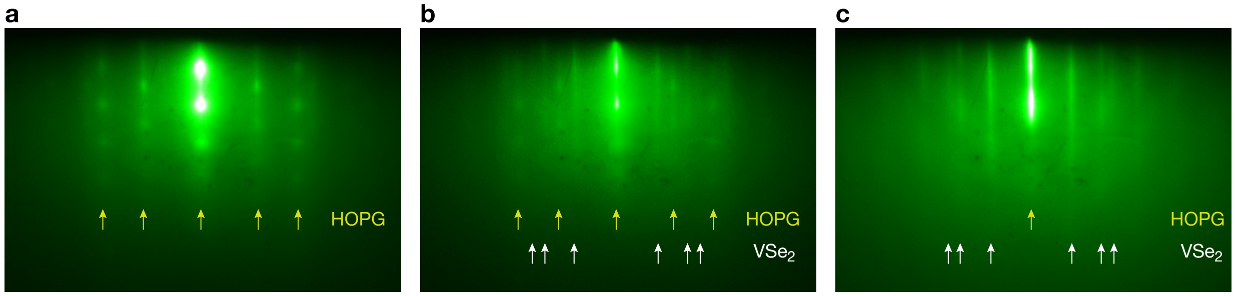

Fig. S3: Reflection high-energy electron diffraction (RHEED) pattern during the growth of monolayer VSe_2. **a**, Before the growth, RHEED pattern of the HOPG substrate. **b**, During the growth. VSe_2 stripes start appearing. **c**, End of growth. VSe_2 now has a high coverage, HOPG's pattern has diminished.

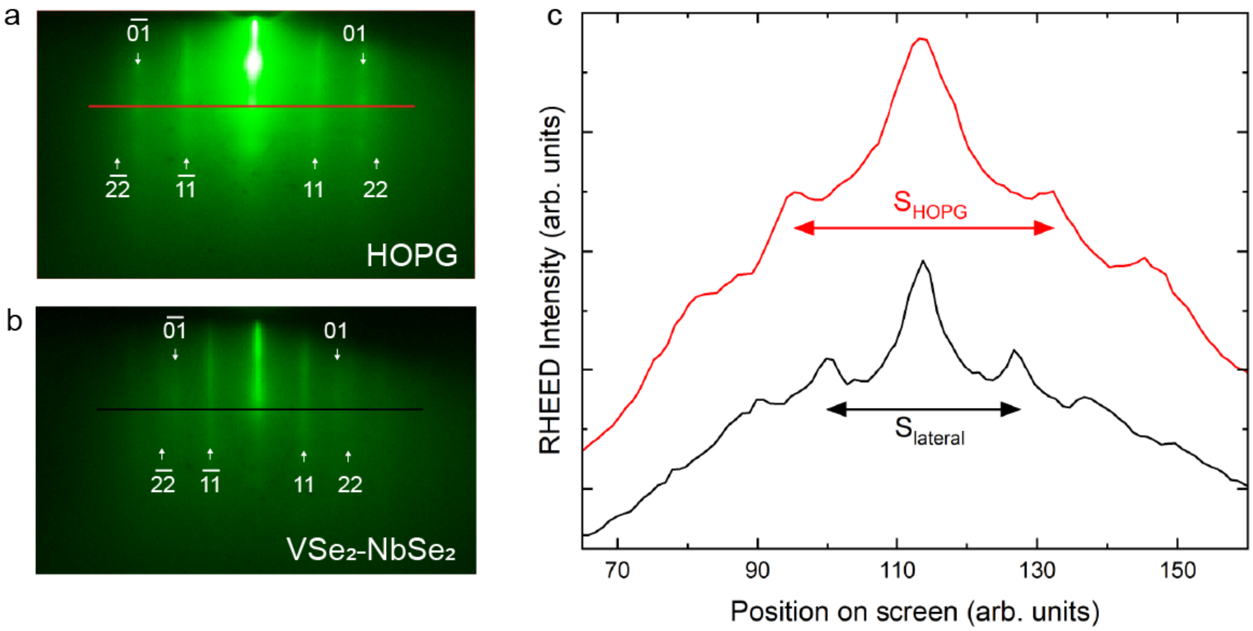

Fig. S4: An example of the determination of the lattice constant from the RHEED pattern. **a**, RHEED pattern of HOPG. **b**, RHEED pattern of the lateral VSe_2–$NbSe_2$ heterostructure. **c**, RHEED intensity line profiles for HOPG (red) and the heterostructure (black) with the streak distances S marked. The corresponding line profile positions are shown in panels **a** and **b**. From the measured streak distances the lattice constant for the heterostructure can be calculated as $(S_{HOPG}/S_{lateral}) \cdot a_{HOPG}$ where a_{HOPG} is the lattice constant of HOPG (2.46 Å).

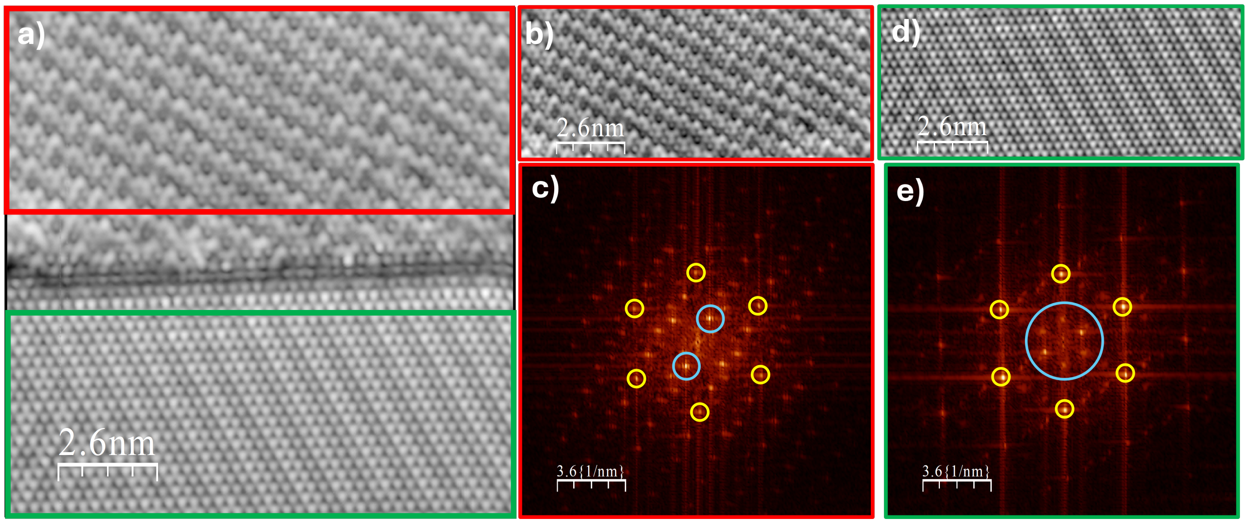

Fig. S5: FFT analysis. **a**, STM image of a 13×13 nm^2 region where an interface can be observed. **b**, Zoom in the VSe_2 part of the heterostructure. **c**, Corresponding FFT of panel b, where yellow circles mark the 1×1 lattice and the blue circles the CDW spots. **d** Zoom in the $NbSe_2$ part of the heterostructure. **e**, Corresponding FFT of panel d, where yellow circles mark the 1×1 lattice and the blue circle is around the 6 spots related with the 3×3 the CDW.

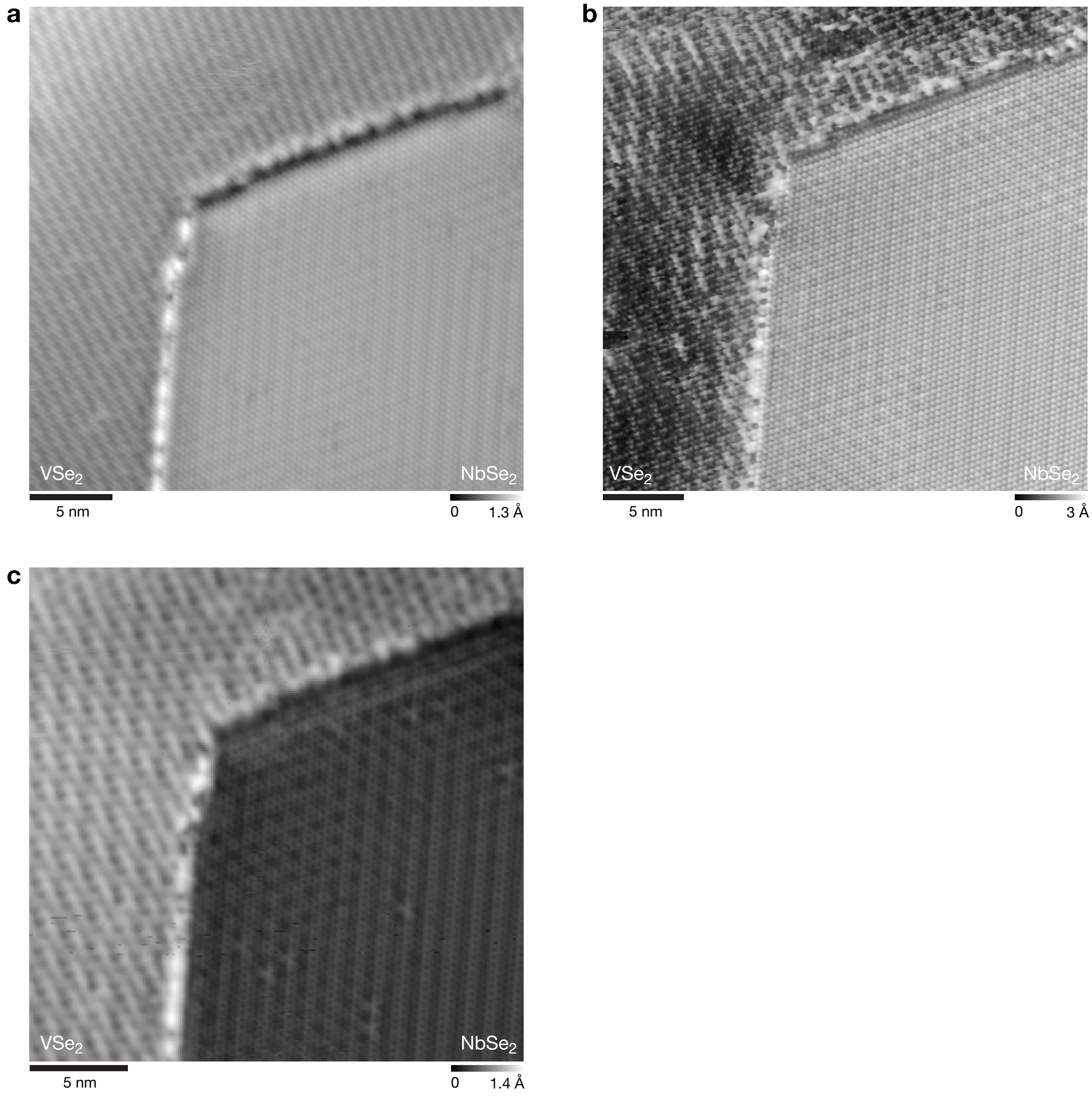

Fig. S6: Two adjacent lateral heterostructures at corner of a $NbSe_2$ island (on A-side). The interfaces shows zigzag morphology, with one of them having bright and and the other dark contrast. The intrinsic CDWs of both materials extend right up to the interface; neither the commensurate CDW of 1H-$NbSe_2$ nor the incommensurate CDW of 1T-VSe_2 extends into the other material. The length of the straight interface sections extend up to $\sim$ 20 nm. **a**, STM topography image of Fig. 2**b**, **i**, **j** and Fig. 3**h**–**j** (V_s=−1 V, I_t= 100 pA). **b**, STM image of the same area with atomic resolution (V_s=−10 mV, I_t= 790 pA). **c**, STM image of the same area showing the charge density waves (V_s=−201 mV, I_t= 50 pA).

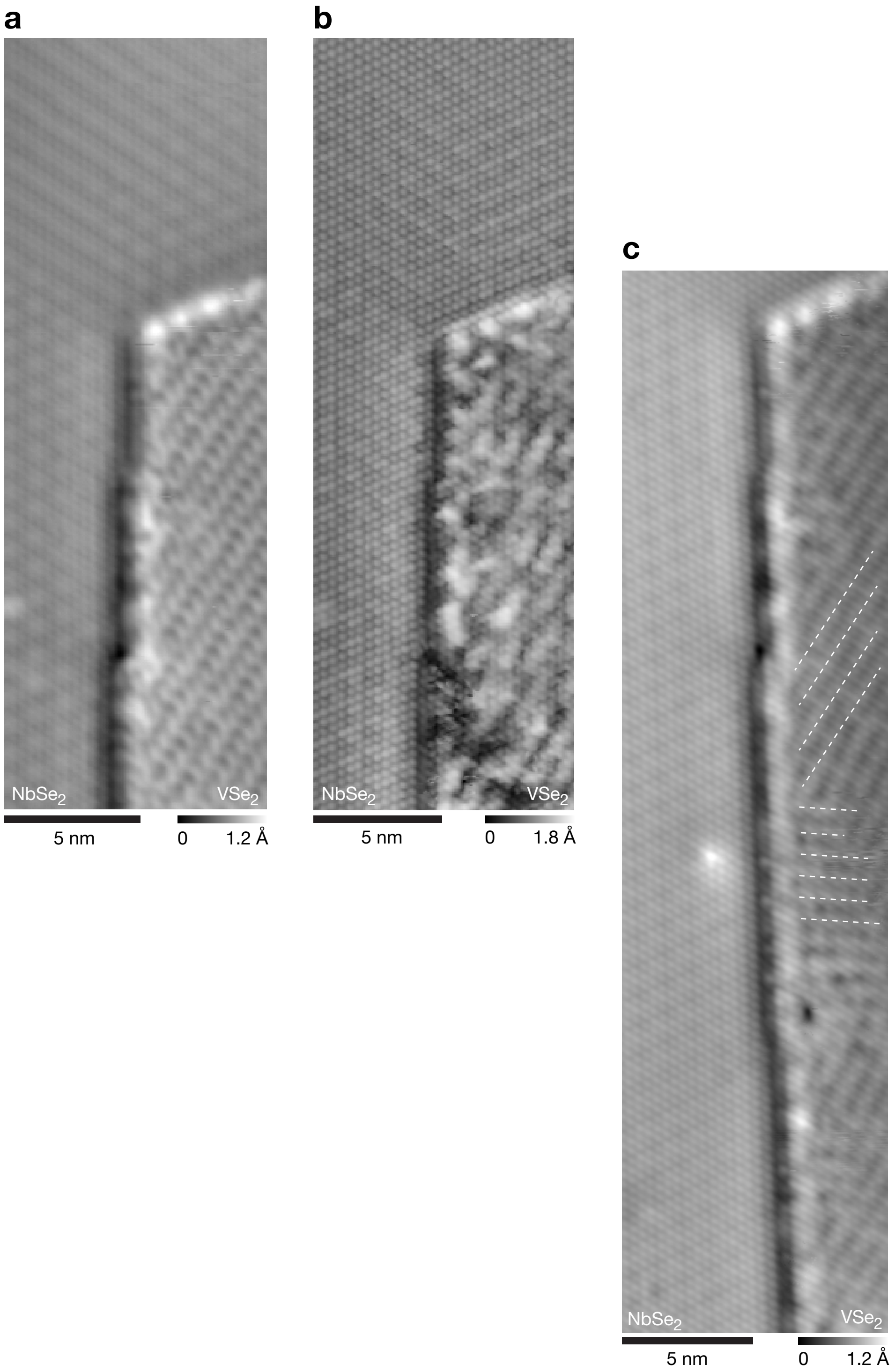

Fig. S7: Additional STM topography images of two adjacent lateral heterostructures across a corner of a $NbSe_2$ island, on A-side. Panel **a**–**c** are aligned with the same position of heterostructures. **a**, STM topography image (V_s=−1 V, I_t= 100 pA). **b**, STM topography image with atomic resolution (V_s=−10 mV, I_t= 1 nA). **c**, STM topography image. The length of the straight interface section extends up to ∼ 40 nm. In the middle part of the dark interface, although the CDW of 1T-VSe_2 changes its direction (indicated with dashed lines), the dark contrast of the interface does not change. (V_s=−1 V, I_t= 100 pA).

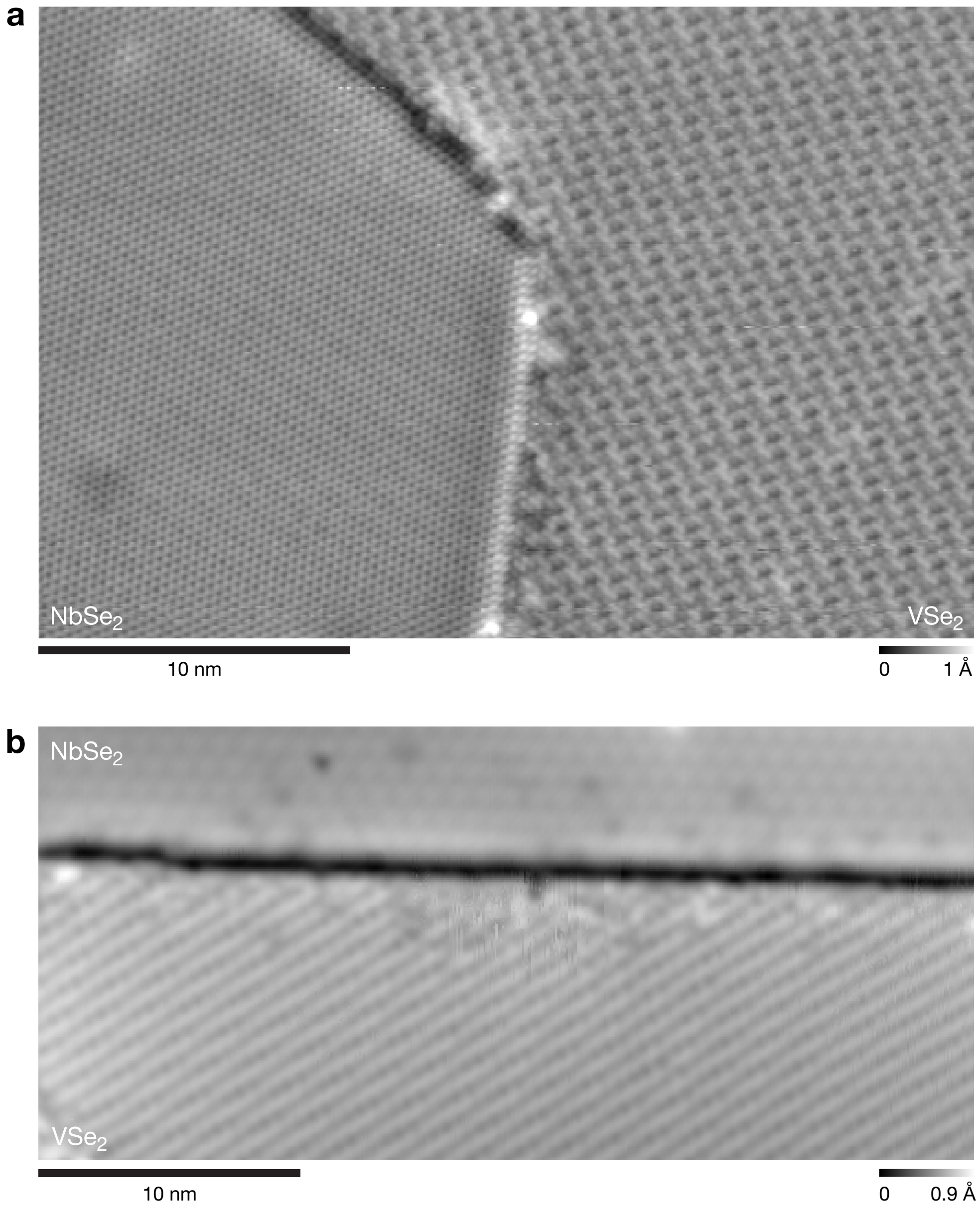

Fig. S8: **a**, Additional STM topography image of lateral heterostructures across a corner of a $NbSe_2$ island, on A-side. (V_s=−1.503 V, I_t=31 pA). **b**, Additional STM topography image of a long interface $\sim$ 36 nm (V_s=+1.36 V, I_t=90 pA).

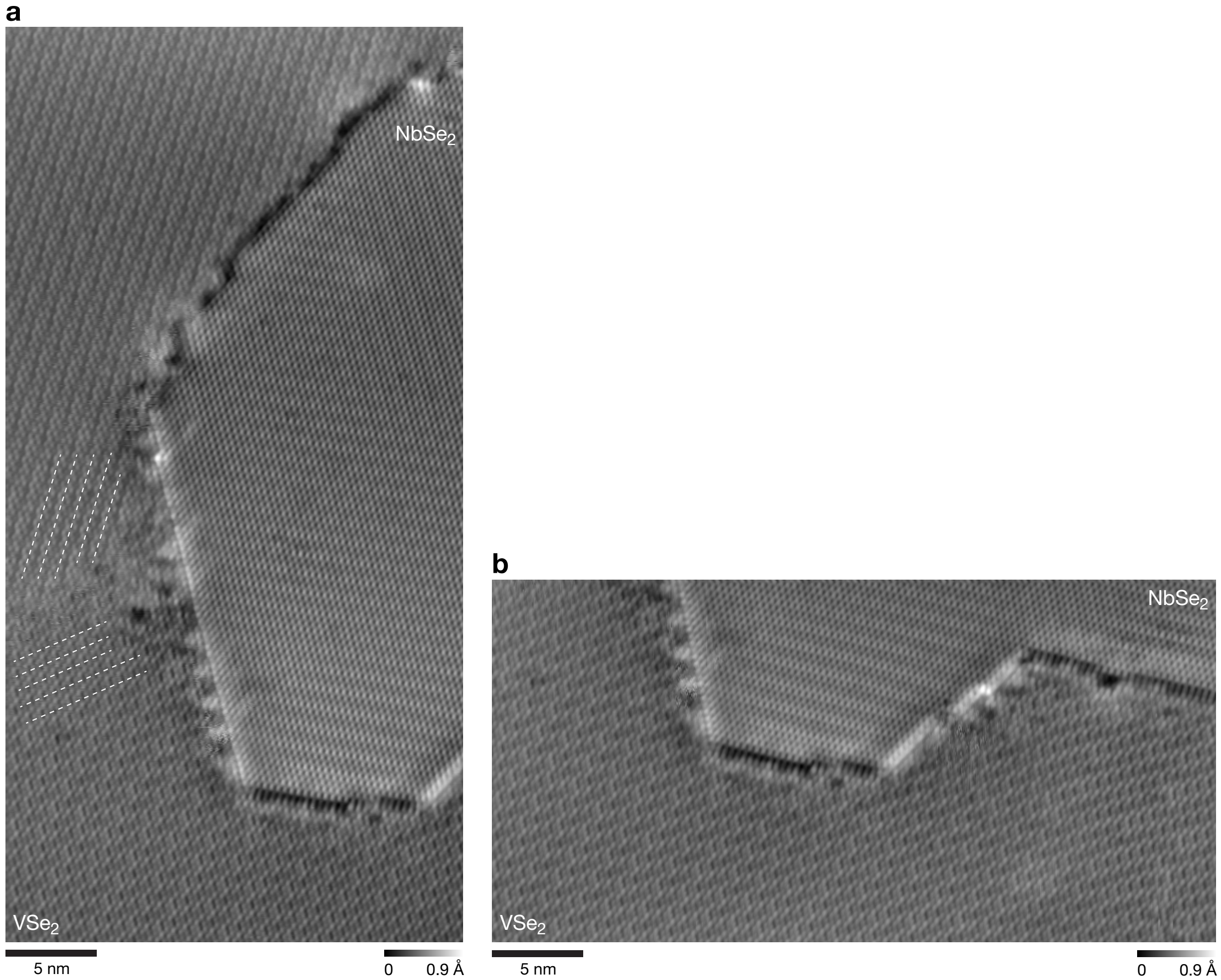

Fig. S9: Adjacent lateral heterostructures across corners of a $NbSe_2$ island, on B-side. The interfaces shows parallel morphology, and bright and dark contrast appears alternatingly at adjacent edges. **a**, STM topography image of Fig. 2**d**, **l**. Along the bright interface, in the middle, the CDW of 1T-VSe_2 changes direction (indicated with dashed lines) but the bright contrast doesn't change. (V_s=−1 V, I_t= 100 pA). **b**, Additional STM image of the same area with an extended range in the x–direction.(V_s=−1 V, I_t= 100 pA).

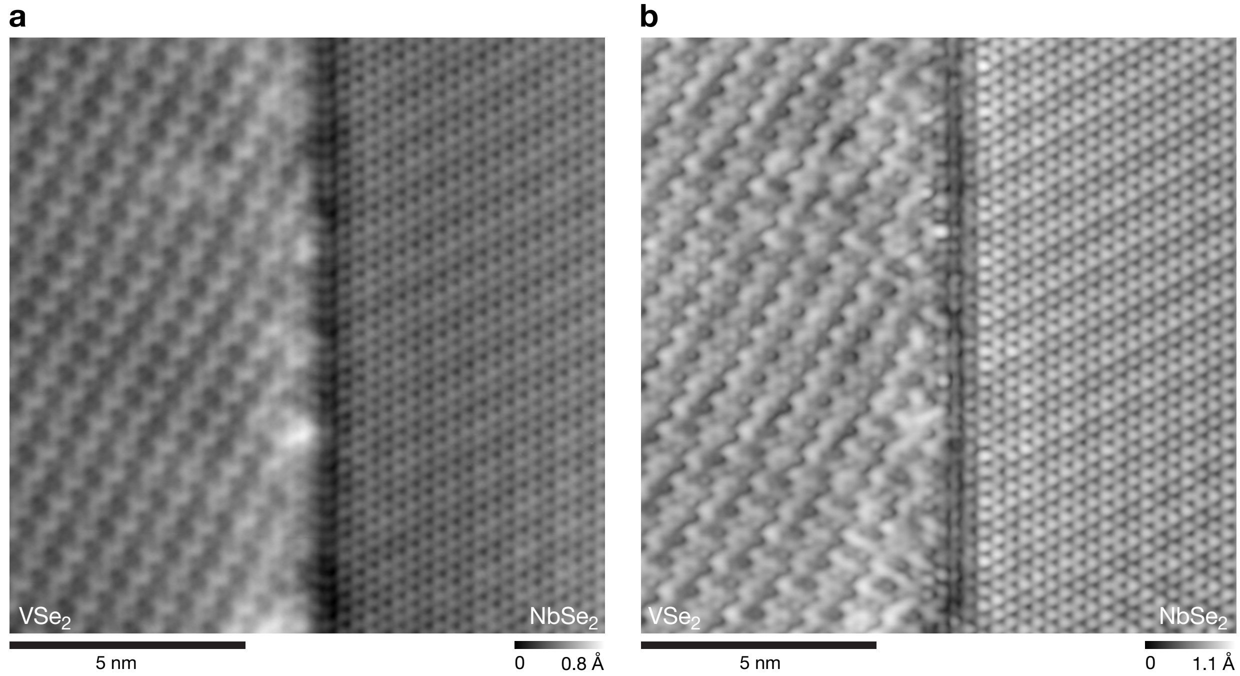

Fig. S10: VSe_5—$NbSe_6$ lateral heterostructure, on B-side. **a**, STM topography image of the interface shown in Fig. 2**k**. (V_s=−0.99 V, I_t= 50 pA). **b**, Additional STM image of the same area (V_s=−50 mV, I_t= 200 pA).

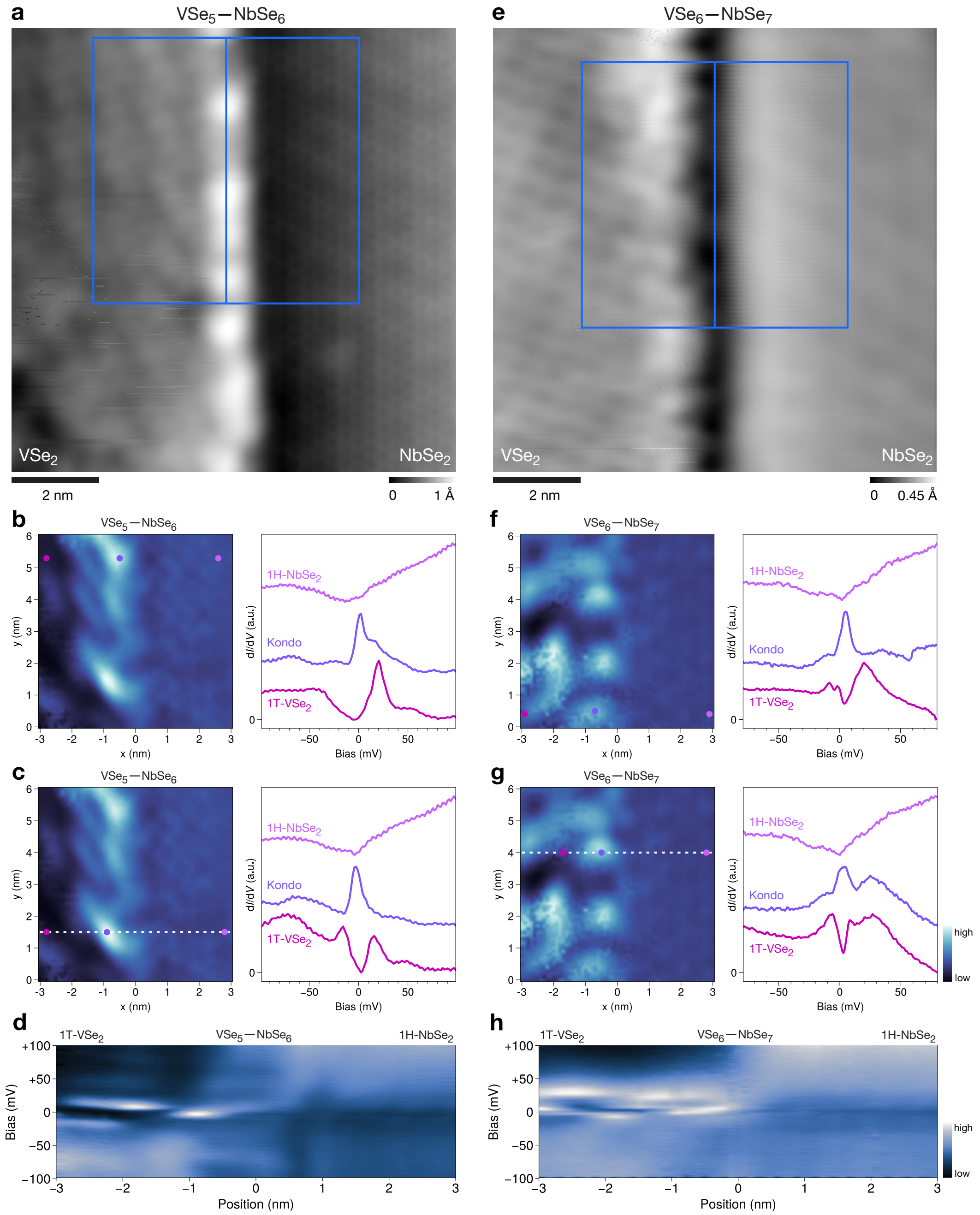

Fig. S11: Signatures of Kondo resonances in a side-coupled geometry. **a**, **e**, STM topography of VSe_5—$NbSe_6$ (Fig. 4 and Fig. 3**g**) and VSe_6—$NbSe_7$ interfaces (Fig. 4). Blue square frames indicate the dI/dV map area; the middle line indicates the 0 position of x-axis in those dI/dV maps. We do not observe defects or alloying in the mapping area. **b**, **c**, (left) dI/dV map at −0.8 mV of VSe_5—$NbSe_6$ interfaces (V_{mod}=2 mV), and (right) corresponding point dI/dV spectra (positions marked in left panel; spectra are shifted vertically for clarity; point spectra taken from the same dI/dV map). **d**, dI/dV spectra along the line indicated in **c**. **f**, **g**, (left) dI/dV map at 0 mV of VSe_6—$NbSe_7$ interfaces (V_{mod}=2 mV), and (right) corresponding point dI/dV spectra (positions marked in left panel; spectra are shifted vertically for clarity; point spectra taken from the same dI/dV map). **h**, dI/dV spectra along the line indicated in **c**. Scan parameters: **a**, V_s=−0.5 V, I_t=210 pA. **e**, V_s=−1.5 V, I_t=380 pA.

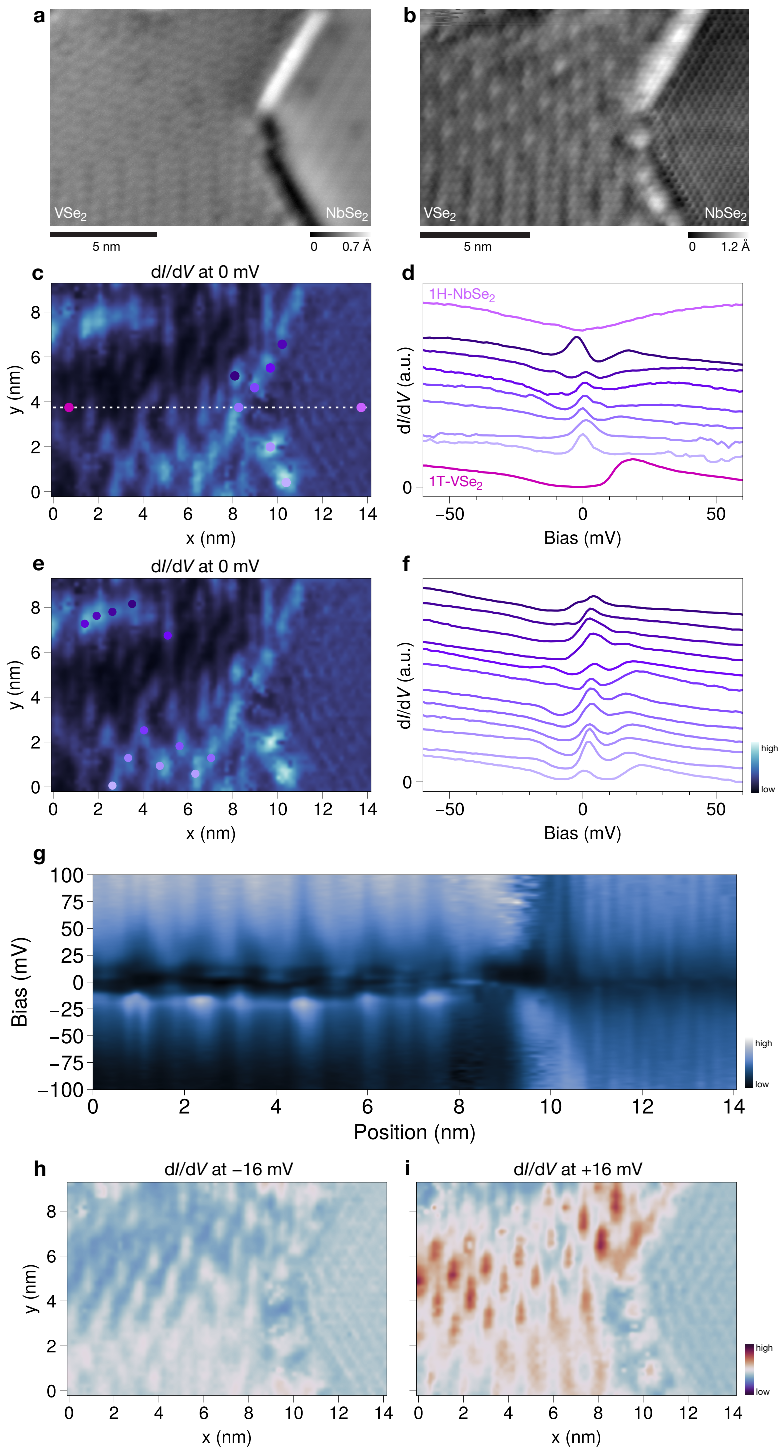

Fig. S12: Two lateral heterostructures and Kondo resonances in a side-coupled geometry. **a**, STM topography. V_s=+1.5 V, I_t=80 pA. **b**, STM topography with atomic resolution. V_s=−0.1 V, I_t= 79 pA. **c**, dI/dV map at 0 mV (V_{mod}=2 mV). **d**, dI/dV spectra of corresponding points in **c**, with points in 1T-VSe_2 and 1H-$NbSe_2$ (spectra are shifted vertically for clarity). **e**, dI/dV map at 0 mV (V_{mod}=2 mV) (same data as in panel c). Signatures of Kondo resonances can be found in VSe_2 up to ∼8 nm away from the interfaces. **f**, dI/dV spectra of corresponding points in **e** (spectra are shifted vertically for clarity). **g**, dI/dV spectra along the line indicated in **c**. **h** and **i**, Contrast inversion of a dI/dV map of these heterostructures at ±16 mV.

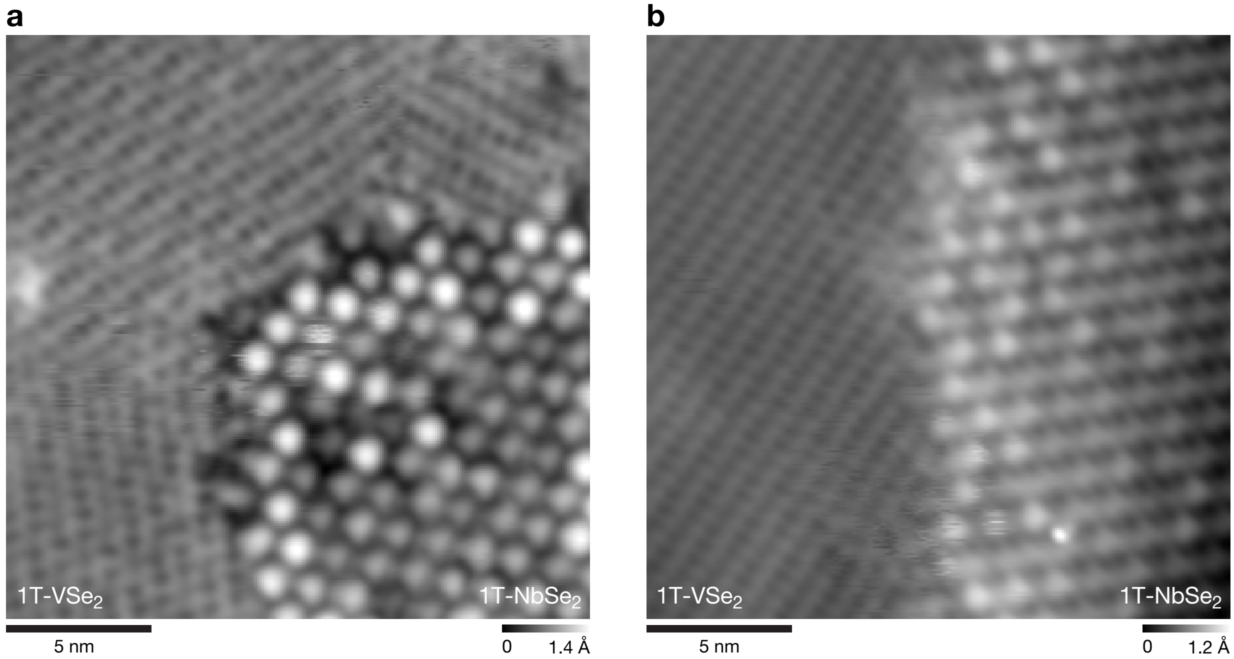

Fig. S13: Additional STM topography images of lateral heterostructures of 1T-VSe_2 and 1T-$NbSe_2$ islands. Also in this case neither CDW extends into the other material. Scan parameters: **a**, V_s=−0.247 V, I_t=62 pA. **b**, V_s=−1.5 V, I_t=150 pA.

DFT calculations

Electronic properties of 2D 1H-$NbSe_2$ and 1T-VSe_2

We tested the effect of the vdw-df2-b86r functional[S8] to the electronic properties of 2D 1H-$NbSe_2$ and 2D 1T-VSe_2. It is known that the vdw-df2-b86r functional with moderate U (e.g. 2 eV) is more appropriate to treat 1T-VSe_2[S9] as opposed to standard functionals such as PBE. Indeed, our DFT results in Figure S14 reveal that the vdw-df2-b86r gives minor quantitative changes in the 1T-VSe_2 band structure, specially at energies close to the Fermi level, while the band structure of 1H-$NbSe_2$ seems to be unaffected. Figure S15 shows the projected density of states (PDOS) and the simulated scanning tunneling spectroscopy (STS), both calculated with the vdw-df2-b86r and PBE functionals for comparison. Apart from very small shifts, the PDOS and simulated STS calculated with different functionals present overall the same phenomenology, specially the simulated STS, which is the most important aspect needed in this work. Thereby, we used the PBE functional with U = 2 eV throughout the calculations with the lateral interface.

Structural and electronic properties of the interfaces

Considering the PBE(U=2 eV) DFT setup defined in the previous section, we performed full relaxation and total energy calculations of different interface structures based on the edge scenarios I and II for the $NbSe_2$ island shown in Figure S16a. Given the nature of the edges in the $NbSe_2$ islands, the edges I and II can coexist in the same unit cell, for example, in the lateral heterostructure shown in Figure S16b, and four $NbSe_2$/VSe_2 interface candidates that can grow in registry were created based on the coordination number of either Nb or V atoms at the interface: I-$NbSe_6$/VSe_5, I-$NbSe_5$/VSe_6, II-$NbSe_6$/VSe_7 and II-$NbSe_7$/VSe_6 (Figures S16c-f, respectively). Notice that all four interfaces present the zig-zag pattern (top orange Se atoms) and parallel pattern (bottom yellow Se atoms) simultaneously. In order to investigate which interfaces in the lateral heterostructure are more energetically favorable

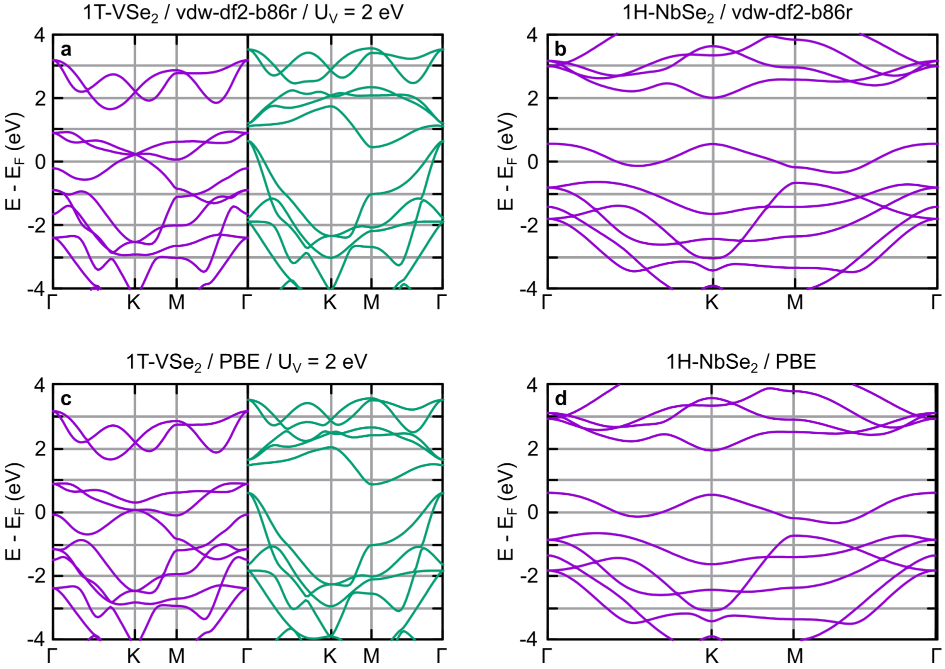

Fig. S14: DFT-calculated band structures of 2D 1H-$NbSe_2$ and 2D 1T-VSe_2 obtained with different functionals

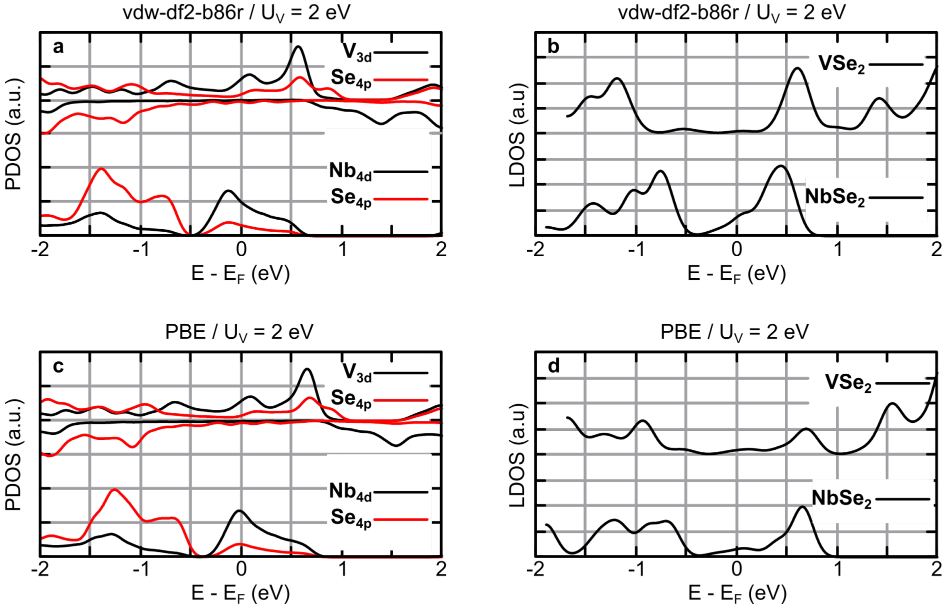

Fig. S15: DFT-calculated PDOS and simulated STS calculated by integrating the local density of states (LDOS) at a constant height of 3 Å over the topmost atom of each structure.

among the structures proposed here, we compared the total energy of structures that differ by only one of the interfaces, thus the total number of atoms will be the same. For example, by keeping the same type of interface I, we were able to calculate and compare the total

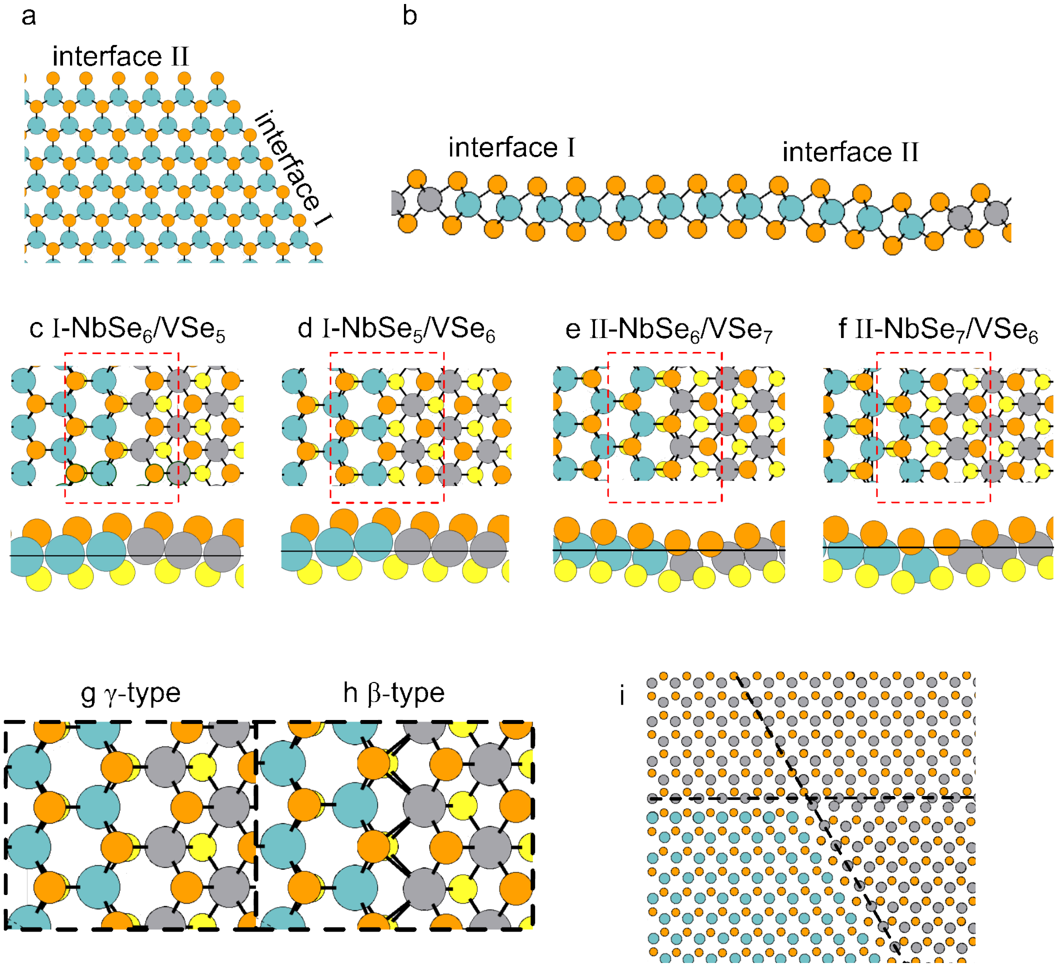

Fig. S16: a: top view of an $NbSe_2$ island showing two possible edge configurations. b: Side view of an example of lateral heterostructures. c-f: Proposed interfaces considered based on the coordination number of the Nb and V atoms at the lateral interface (A-side). g and h: Examples of the γ and β types of interfaces. i: Example of a tentative overlapping of a heterostructure with coexisting two β-type interface, showing that it cannot grow in registry (only Nb, V and top Se atoms are shown).

energies of the two types of interface II shown in Figure S16e and f, and the same procedure goes by keeping interface II as well. Our calculations show that among the type I interfaces, the I-$NbSe_6/VSe_5$ is more stable than the I-$NbSe_5/VSe_6$ by 0.2 eV, while among type II the II-$NbSe_7/VSe_6$ is more stable than II-$NbSe_6/VSe_7$ by 0.4 eV. Even though the total energies are affected by a twist of the unit cell that occurs in order to keep two interfaces, the energy spent in this deformation is two orders of magnitude smaller than the energy differences obtained here, thus not creating any artificial stability. We also stress that all interfaces

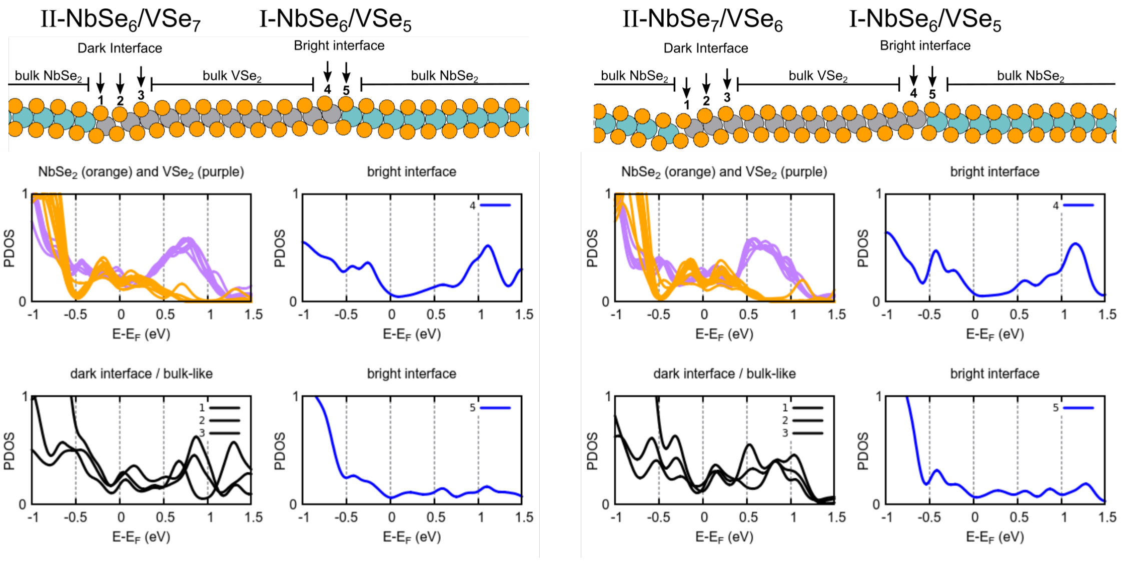

Fig. S17: DFT-calculated PDOS on the p_z orbitals of interface region Se atoms (A-side).

considered here are of the $\gamma-$type, whereas interfaces of the $\beta-$type do not grow in registry and would induce strong strain and reorganization of the interfaces, which is not compatible to the experimental results (see Figures S16g, h and i). Mixing of γ and β-types also cannot grow in registry. A simple way to distinguish the β- and γ-type interfaces is if transition metal atoms are aligned or not: for β-type, metal atoms are aligned in parallel, while for γ-type metal atoms are in a zigzag configuration at the interfaces.[S10]

The DFT-calculated energies indicate that the islands where the interfaces I-$NbSe_6/VSe_5$ and II-$NbSe_7/VSe_6$ coexist are preferable. However, even though the I-$NbSe_6/VSe_5$ is already expected to occur due to the stability of the edge I Nb having coordination six, it is very difficult to rule out the occurrence of the II-$NbSe_6/VSe_7$ interface just based on our STM and LDOS results. We then proceeded to investigate two lateral heterostructures shown in Figure S17, which differ only by the interface II. Additionally, the atomic contributions to the different STM signals and LDOS in the bright and dark interfaces are elucidated in more details in Figure S17, showing the DFT-calculated PDOS on the p_z orbitals of each top Se atom (A-side structure) of both lateral heterostructures. These are the atoms that contribute the most to the tunneling current, and will directly influence the contrast in the STM and LDOS either due to an electronic effect, for example having lower or higher density

of states, or due to the undulation of the relaxed structure. In both lateral heterostructures, the Se atom belonging to VSe_2 immediately at the bright interface has a significantly shifted PDOS compared to its surrounding Se atoms, which is then responsible to the strong bright features observed at -0.4 eV and 1.2 eV in the LDOS in the main text Figure 3f. Aside from a shift in energy, both features are consistent with the ones observed in the experiment at the bright interface. For the dark interface of the lateral heterostructure at the left of Figure S17, we observed a small attenuation of the PDOS of the Se at the interface belonging to the VSe_2, which are much lower in height than the other atoms in the bulk area. We then attribute in this case the darker contrast to both electronic and geometric effects. On the other hand, no attenuation is observed in the heterostructure at the right side of Figure S17, meaning that only the undulation is responsible to the dark contrast observed.

Figure S18 shows the alternative lateral heterostructure where only interface II is different from the one presented in the main text. The simulated STM images for the two types of interface II structures (VSe_7—$NbSe_6$ / VSe_6—$NbSe_7$) are quite similar but as mentioned before VSe_7—$NbSe_6$ was found to be less stable based on DFT.

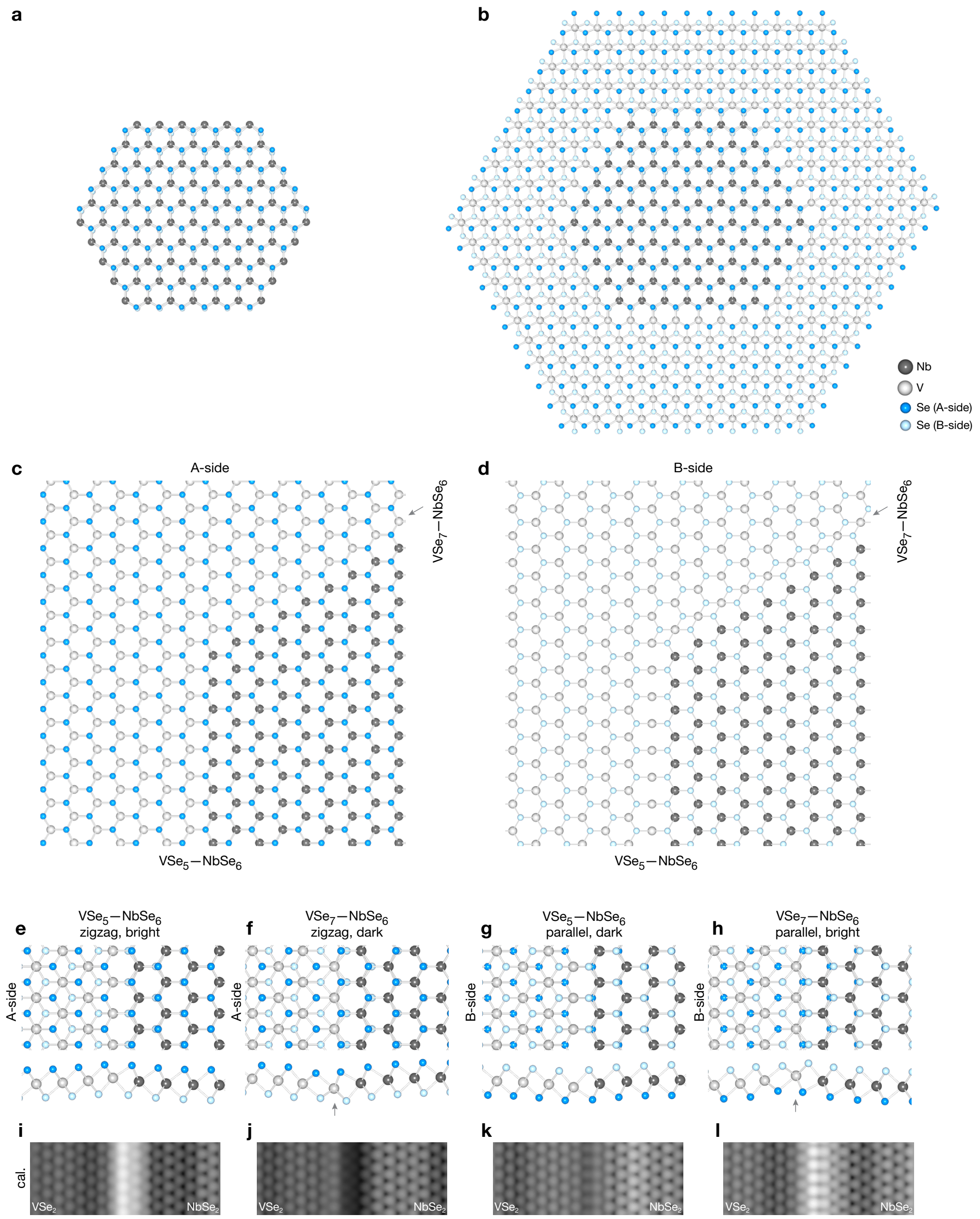

Fig. S18: Alternative lateral heterostructure geometry with VSe_5—$NbSe_6$ and VSe_7—$NbSe_6$ interfaces. Compared to results reported in main text, the structure of the VSe_5—$NbSe_6$ interface remains unaltered. The main structural difference between VSe_7—$NbSe_6$ and VSe_6—$NbSe_7$ is that a line of transition metal atoms at the interface is replaced from V atoms to Nb atoms (indicated by the grey arrows in panels **c**, **d**, **f** and **h**). DFT calculations suggest VSe_6—$NbSe_7$ is more stable. **a**, **b**, Schematics of the formation of a lateral heterostructure in this geometry: VSe_2 and $NbSe_2$ can also grow in-registry with VSe_5—$NbSe_6$ and VSe_7—$NbSe_6$ interfaces. **c**, **d**, Zoom-in schematic of two interfaces with views from A-side (with A-side Se atoms only) and B-side (with B-side Se atoms only), respectively. Grey arrows indicate the difference between VSe_7—$NbSe_6$ and VSe_6—$NbSe_7$ (schematic structures without relaxation). **e**–**h**, Top view and side view of the these lateral heterostructures of VSe_5—$NbSe_6$ and VSe_7—$NbSe_6$, and **i**–**l**, their corresponding calculated STM images (V_s=−0.5 V) (calculated structures with relaxation). Grey arrows indicate the difference between VSe_7—$NbSe_6$ and VSe_6—$NbSe_7$.

References

(S1) Liu, Z.-L.; Wu, X.; Shao, Y.; Qi, J.; Cao, Y.; Huang, L.; Liu, C.; Wang, J.-O.; Zheng, Q.; Zhu, Z.-L.; Ibrahim, K.; Wang, Y.-L.; Gao, H.-J. Epitaxially Grown Monolayer VSe_2 : An Air-Stable Magnetic Two-Dimensional Material with Low Work Function at Edges. *Science Bulletin* **2018**, *63*, 419–425.

(S2) Akber, H.; Shan, H.; Mao, Y.; Yao, J.; Zhai, X.; Zhao, A. Nonreciprocal Charge-Density-Wave Proximity Effect in a Lateral Heterojunction of $NbSe_2/TiSe_2$. *Appl. Phys. Lett.* **2024**, *124*, 071602.

(S3) Zhang, Q.; Fan, J.; Zhang, T.; Wang, J.; Hao, X.; Xie, Y.-M.; Huang, Z.; Chen, Y.; Liu, M.; Jia, L.; Yang, H.; Liu, L.; Huang, H.; Zhang, Y.; Duan, W.; Wang, Y. Visualization of Edge-Modulated Charge-Density-Wave Orders in Monolayer Transition-Metal-Dichalcogenide Metal. *Commun. Phys.* **2022**, *5*, 117.

(S4) Lozovoy, K. A.; Dirko, V. V.; Kukenov, O. I.; Sokolov, A. S.; Krukovskii, K. V.; Snegerev, M. S.; Borisov, A. V.; Kistenev, Y. V.; Kokhanenko, A. P. RHEED Study of the Epitaxial Growth of Silicon and Germanium on Highly Oriented Pyrolytic Graphite. *C* **2024**, *10*, 36.

(S5) Soumyanarayanan, A.; Yee, M. M.; He, Y.; van Wezel, J.; Rahn, D. J.; Rossnagel, K.; Hudson, E. W.; Norman, M. R.; Hoffman, J. E. Quantum Phase Transition from Triangular to Stripe Charge Order in $NbSe_2$. *Proc. Natl. Acad. Sci* **2013**, *110*, 1623–1627.

(S6) Flicker, F.; van Wezel, J. Charge Ordering Geometries in Uniaxially Strained $NbSe_2$. *Phys. Rev. B* **2015**, *92*, 201103.

(S7) Cossu, F.; Palotás, K.; Sarkar, S.; Di Marco, I.; Akbari, A. Strain-Induced Stripe Phase in Charge-Ordered Single Layer $NbSe_2$. *NPG Asia Mater.* **2020**, *12*, 24.

(S8) Hamada, I. van der Waals Density Functional Made Accurate. *Phys. Rev. B* **2014**, *89*, 121103.

(S9) Kezilebieke, S.; Huda, M. N.; Dreher, P.; Manninen, I.; Zhou, Y.; Sainio, J.; Mansell, R.; Ugeda, M. M.; van Dijken, S.; Komsa, H.-P.; Liljeroth, P. Electronic and Magnetic Characterization of Epitaxial VSe_2 Monolayers on Superconducting $NbSe_2$. *Commun. Phys.* **2020**, *3*, 116.

(S10) Lin, Y. C.; Dumcenco, D. O.; Huang, Y. S.; Suenaga, K. Atomic Mechanism of the Semiconducting-to-Metallic Phase Transition in Single-Layered MoS_2. *Nat. Nanotechnol.* **2014**, *9*, 391–396.
